# Quercetin activates energy expenditure to combat metabolic syndrome through modulating gut microbiota-bile acids crosstalk in mice

**DOI:** 10.1080/19490976.2024.2390136

**Published:** 2024-08-20

**Authors:** Xiaoqiang Zhu, Xiaojuan Dai, Lijun Zhao, Jing Li, Yanhong Zhu, Wenjuan He, Xinlei Guan, Tao Wu, Li Liu, Hongping Song, Liang Lei

**Affiliations:** a Central Laboratory, Wuhan Fourth Hospital, Wuhan, China; b Department of Pharmacy, Wuhan Fourth Hospital, Wuhan, China; c National Engineering Research Center for Nanomedicine, College of Life Science and Technology, Huazhong University of Science and Technology, Wuhan, China; d Department of Gastroenterology, Wuhan Fourth Hospital, Wuhan, China; e Hubei Jiangxia Laboratory, Wuhan, China; f Pharmaceutical Department, Hubei Cancer Hospital, Tongji Medical College, Huazhong University of Science and Technology, Wuhan, China

**Keywords:** Quercetin, metabolic syndrome, energy metabolism, gut microbiota, bile acids

## Abstract

Abdominal obesity-related metabolic syndrome (MetS) has emerged as a significant global public health issue that affects human health. Flavonoids, such as quercetin, have been reported to exert obvious anti-obesity and lipid-lowering effects in both humans and animal models. However, the precise underlying mechanism remains elusive. In this study, we investigated the potential roles of gut microbiota-bile acids (BAs) interactions in quercetin-induced anti-obesity effects and metabolic benefits. Oral administration of quercetin significantly enhanced energy metabolism through activating thermogenesis of brown adipose tissues (BAT) and browning of white adipose tissues (WAT), thus mitigating metabolic dysfunctions in an abdominal obesity-related MetS mouse model. Further mechanistic studies demonstrated that quercetin treatment substantially promoted the generation of non-12α-hydroxylated BAs (non-12OH BAs), particularly ursodeoxycholic acid (UDCA) and lithocholic acid (LCA), in serum via regulating the overall structure of gut microbiota and enriching *Lactobacillus*. High level of non-12OH BAs bind to Takeda G protein-coupled receptor 5 (TGR5) on adipocytes to stimulate thermogenesis. Remarkably, fecal microbiota transplantation (FMT) from quercetin-treated mice replicated the effects of quercetin on non-12OH BAs generation and energy expenditure, which suggested gut microbiota reshape and concomitant BAs regulation were responsible for the benefits on energy metabolism of quercetin in the MetS mouse model. Our findings not only highlighted the critical role of gut microbiota-BAs crosstalk in mediating quercetin-induced energy expenditure, but also enriched the pharmacological mechanisms of quercetin in ameliorating MetS-related diseases.

## Graphical abstract

**Figure:**
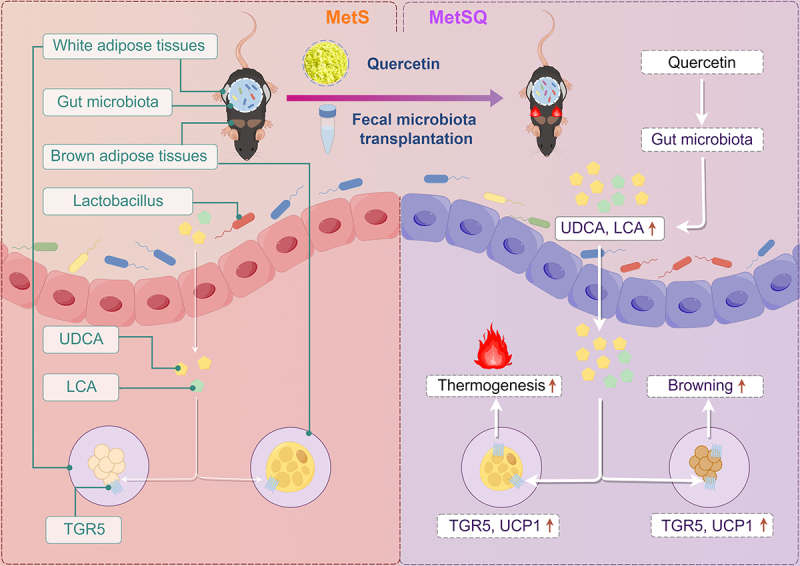

## 1. Introduction

Metabolic syndrome (MetS) is acknowledged as a progressive chronic pathological condition characterized by manifestations including abdominal obesity, dyslipidemia, and hyperglycemia, which obviously elevate the risks of adverse cardiovascular events and colorectal cancer.^1^ Recent epidemiological evidence has demonstrated a significant surge in global incidence rates of MetS. Statistics revealed that the prevalence of MetS in adults was 34.7% in the United States and 33.9% in China, respectively, with increasing growth rate due to escalating morbidity of diabetes and obesity over recent decades.^2^ The development of MetS is influenced by a combination of genetic and environmental factors, resulting in an imbalance between energy intake and expenditure.^3^ Therefore, implementing strategies to reduce energy intake and/or increase energy expenditure is considered a promising strategy for treating obesity-related MetS.^4^ Several studies have demonstrated that activating brown adipose tissues (BAT) and inducing browning of white adipose tissues (WAT) enhanced the thermogenic activity mediated by mitochondrial uncoupling protein 1 (UCP1), leading to increased energy expenditure and improvement of obesity-related metabolic dysfunctions. ^4–6^

The relationship between gut microbiota and energy balance in metabolic diseases has garnered widespread attention.^7^ Some studies have shown that gut microbiota can regulate the thermogenic process of adipose tissues.^8^ Cold stimulation or intermittent fasting could improve metabolic disorders by promoting WAT browning via regulating intestinal microbes, while depletion of gut microbiota decreased thermogenic capacity.^9–11^ Therefore, targeting intestinal bacteria to promote adaptive thermogenesis has been proposed as a novel strategy to effectively combat metabolic diseases. Previous studies have shown that fecal microbiota transplantation (FMT), probiotics, or prebiotics could modulate gut microbiota composition to enhance energy metabolism.^12–14^ Certain gut bacterial species, such as *Akkermansia muciniphila* and *Parabacteroides distasonis*, have been reported to promote thermogenesis.^15,16^ The beneficial effects of gut microbiota on energy expenditure are largely determined by gut bacterial metabolites, such as non-12α-hydroxylated bile acids (non-12OH BAs).^17^ Elevated levels of non-12OH BAs have been proven to induce obesity-resistant effects through Takeda G protein-coupled receptor 5 (TGR5)-mediated UCP1 induction and BAT activation.^18–20^ Thus, identifying new agents targeting gut bacteria and their metabolic pathways to enhance thermogenesis is of great significance to treat abdominal obesity-related MetS.

Quercetin (3,3′,4′,5,7-pentahydroxyflavone), a naturally occurring flavonoid found in fruits and vegetables, exhibits multiple biological functions including anti-inflammatory, anti-oxidant, and anti-obesity activities.^21^ Evidences from animal studies suggested quercetin administration ameliorated high-fat diet (HFD)-induced obesity-related metabolic disorders through regulating the structure of gut microbiota.^22^ Furthermore, a few studies have shown that quercetin could enhance the browning of white adipocytes *in vitro* and *in vivo*.^23–25^ However, the precise underlying mechanism remains unclear. More importantly, it has not been investigated whether the gut microbiota and its metabolites are involved.

In this study, we aimed to explore the contribution of interactions between gut microbiota and bile acids (BAs) to the beneficial effects of quercetin on abdominal obesity-related MetS using 16S rDNA sequencing, BAs-targeted metabolomics, and FMT techniques. Our results revealed that quercetin-mediated gut microbiota remodeling was responsible for its anti-MetS effect, and we identified non-12OH BAs as important gut microbiota-derived metabolites involved in this process, specifically ursodeoxycholic acid (UDCA) and lithocholic acid (LCA), which promoted browning of WAT and thermogenic activity of BAT in response to quercetin treatment. These findings provide new insights into how quercetin can improve metabolic diseases by targeting gut microbiota and regulating BAs metabolism, and contribute to our understanding of the pharmacological mechanisms underlying quercetin’s therapeutic potential for treating metabolic diseases.

## 2. Materials and methods

### 2.1. Animals

The specific pathogen-free C57BL/6J mice were purchased from Hubei Center for Disease Control and Prevention, China (Wuhan, China). Mice were housed under controlled conditions with a 12-hour light–dark cycle at a constant temperature of 22 ± 2°C and humidity of 55 ± 5%. All animal experiments conducted in this study received approval from the Institutional Animal Care and Use Committee at Tongji Medical College, Huazhong University of Science and Technology. The abdominal obesity-related MetS mouse model was selected due to the pivotal role of abdominal obesity in MetS, which was constructed as previously described with minor modifications.^26,27^ Briefly, neonatal mice were subcutaneously injected with saline (Con mice) or monosodium glutamate (MetS mice) at a dose of 3 mg/g body weight from day 2 to 8 after birth daily. The male mice were provided free access to standard normal chow diet (WQJX Bio-Technology, China) and water. At weeks 12 to 18, the MetS group was further divided into two subgroups: MetS group and MetSQ group. The MetSQ mice were gavaged with 50 mg/kg/day quercetin (Push Bio-Technology, China) dissolved in 0.15% carboxymethylcellulose sodium (Aladdin, China), while Con and MetS mice were just administrated with an equal volume (100 μL/10 g body weight) of 0.15% carboxymethylcellulose sodium. Body weight and food intake were recorded weekly from week 12 to 18. The waist circumference was recorded at the end of the experiment. At week 18, fresh feces pellets were collected in a sterile environment, then all mice were sacrificed, and liver and adipose tissues were stripped. The liver weight and fat mass were measured. All samples were stored at −80°C.

### 2.2. Blood biochemical analysis and measurement of hepatic lipid profiles

At the end of the experiments, blood samples were collected and centrifuged (3000 rpm, 10 min) for obtaining sera. After dilution with phosphate buffered saline (PBS), the serum samples were used to measure the levels of total cholesterol (TC), triglycerides (TG), low-density lipoprotein cholesterol (LDL-C), high-density lipoprotein cholesterol (HDL-C), free fatty acids (FFA), fasting blood glucose (FBG), total bile acid (TBA), aspartate aminotransferase (AST), and alanine aminotransferase (ALT) using an automatic biochemical analyzer (Mindary, China). Hepatic TC and TG levels were measured using commercial enzyme kits (Nanjing Jiancheng Bioengineering Research Institute, China) normalized by liver tissue weight.

### 2.3. Histology

Adipose tissues were fixed in 4% paraformaldehyde for 12 h, embedded in paraffin, and then stained with hematoxylin and eosin (H&E) to detect morphological changes in adipose tissues. Liver tissues were stained with Oil Red O to observe lipid deposition. Immunohistochemical staining was performed to UCP1 expression in adipose tissues with anti-UCP1 antibody (1:500, Sevicebio, China). Images were acquired by a light microscope (Nikon Eclipse TE2000-U, NIKON, Japan).

### 2.4. Oral glucose tolerance test (OGTT)

At week 18, mice were orally administrated glucose solution at a dose of 2.5 g/kg body weight following 16 h fasting. Blood glucose concentrations were measured using Roche glucometers (Roche, China) by tail bleeding at 0, 30, 60, and 120 min.

### 2.5. Metabolic activity

Mice were placed individually in metabolic cages (Comprehensive Lab Animal Monitoring System, Columbus, USA) at 22°C under a 12 h light–dark cycle with free access to food and water to measure energy expenditure, oxygen consumption (VO_2_), carbon dioxide production (VCO_2_) and activity accounts.

### 2.6. Core temperature and infrared image

Rectal core temperature was assessed using the BAT-12 Microprobe-Thermometer (Physitemp, USA) before and after 12 h cold challenge (16 °C). BAT temperature was measured with an infrared thermal imager (FLIR, USA).

### 2.7. Quantitative real-time PCR (qRT-PCR)

RNA was extracted from cells and tissues with the RNAiso Plus reagent (Takara, Japan). RNA concentration was quantified with NanoDrop 2000 spectrophotomer (Thermo Scientific, USA). The cDNA synthesis was performed utilizing the PrimeScript RT reagent Kit (Takara, Japan). The qRT-PCR reaction was conducted using SYBR Premix Ex TaqTM I reagent Kit (Takara, Japan) with 7500 Real-Time PCR System (Applied Biosystems, USA) as described.^28^ The primer sequences used were listed in Table S1.

### 2.8. Enzyme-linked immunosorbent assay (ELISA)

The weighed adipose tissues were homogenized in PBS with High Throughput Tissue Grinder (Scientz, China). After centrifugation (3000 rpm, 10 min) at 4°C, the supernatant was collected to quantify UCP1 concentrations with commercial ELISA kits (MSKBIO, China) according to the manufacturer’s instructions.

### 2.9. Bile acid (BA) profiles analysis

The composition of serum BAs was analyzed on ultra-high performance liquid chromatography-mass spectrometry as decribed with some modifications.^29^ BAs were extracted from serum as follows: 200 μL of methanol was added to 50 μL of serum, and mixed by vortexing for 2 min. Next, samples were centrifuged at 20,000 g (4°C) for 10 min to collect the supernatant for analysis.

Liquid chromatography (LC) separation was performed using ACQUITY UPLC I-Class ultra-high performance liquid chromatography (Waters, UK) with an ACQUITY UPLC BEH C8 (50 × 2.1 mm, 1.7 μm) column (Waters, UK) with a temperature of 50°C. The mobile phases consisted of 10 mM ammonium acetate in water containing 0.1% ammonia (phase A) and 10 mM ammonium acetate containing 0.1% ammonia in acetonitrile:methanol (70:30) (phage B) at a flow rate of 0.5 mL/min. The elution gradient was performed as below: 0–1 min, 30%–40% B; 1–4 min, 40%–100% B; 4–5 min, 100%–30% B. The sample injection volume was 5 μL. Mass spectrometry (MS) was performed with a QTRAP 6500 + triple quadrupole mass spectrometer (SCIEX, USA) equipped with an ESI probe operated with multiple reaction monitoring (MRM) in negative-ion mode. The ion spray voltage was −4500 V, ion source temperature was 500°C. Data were acquired and processed using the Analyst software (SCIEX, USA).

### 2.10. Cell culture and treatments

The isolation and culture of brown pre-adipocytes were performed as previously described.^30^ In brief, brown adipose tissues isolated from 18-week-old MetS mice were promptly cut into small pieces and then digested with 1 mg/mL type I collagenase (Gibco, USA) for 30 min at 37°C. The digested tissue was filtered through a 100 µm nylon screen and subsequently centrifuged at 2500 rpm for 5 min at 4°C. Cells were seeded in 6-well plates. The differentiation of brown adipocytes was conducted following established methods.^31^ To explore the impact of UDCA on thermogenesis, differentiated mature mouse brown adipocytes were treated with different concentrations of UDCA (Aladdin, China) for 72 h. Then the qRT-PCR was employed to detect the mRNA expression levels of thermogenic genes.

### 2.11. 16S rDNA sequencing and analysis

Fresh stools collected at week 18 were immediately snap-frozen in liquid nitrogen and stored at −80°C. Fecal microbial DNA was extracted and quantified with NanoDrop 2000 spectrophotomer (Thermo Scientific, USA). The V4 hypervariable region of the 16S rDNA was amplified from extracted DNA and assessed with the AxyPrep DNA gel extraction kit, followed by fluorescence quantitation with Qubit 2.0 Fluorometer. The diluted samples were then sequenced on Illumina MiSeq PE250 platforms to generate paired-end reads. After filtering out low-quality reads with default parameters, the consensus sequences were generated with FLASH (v1.2.11) once the two paired-end reads overlapped. The operational taxonomic units (OTUs) were clustered with a 97% similarity threshold using UPARSE, then the tags number of each OTU in each sample was summarized to the OTU abundance table, and the relative abundance of each OTU was calculated. The tags number of each taxonomic rank were summarized in a profiling table for determining relative abundances of taxa at various taxonomic levels. α and β diversity analyses were performed using QIIME (v2.0). The principal coordinate analysis (PCoA) was conducted using R software (v3.1.1). Linear discriminant analysis of effect size (LEfSe) was carried out on ImageGP website (http://www.bic.ac.cn/BIC/#/.) to identify bacterial taxa with LDA scores >2.0 and average relative abundances >0.01%.

### 2.12. FMT

The bacterial suspension was prepared as described.^32^ Briefly, fresh stool samples were collected from 4 age-matched MetS and MetSQ donor mice between 9 and 10 a.m. in the FMT day. The fresh feces were diluted in ice-cold sterile saline (100 mg feces/1 mL saline) and then steeped in the cold saline for 5 min, followed by 10 s vigorous vortex, and was finally centrifuged (800 g, 4 °C) for 3 min. The resulting supernatants were used for the FMT. Prior to FMT, the recipient MetS mice received antibiotics (ABX) drinking (containing 1 mg/mL ampicillin, 1 mg/mL neomycin, 1 mg/mL metronidazole and 0.5 mg/mL vancomycin) for 1 week to deplete gut microbiota. The recipient MetS mice were administrated 200 μL fecal supernatant from either MetS (MetS→MetS group) or MetSQ (MetSQ→MetS group) donor mice by oral gavage three times a week for 6 weeks.

### 2.13. Statistical analysis

The results were expressed as mean ± standard error of the mean (s.e.m). The statistical significance was determined by two-tailed Student’s *t* test for parametric variable comparisons between two groups, and one-way analysis of variance (ANOVA) followed by Tukey’s post hoc test for comparisons among three groups in GraphPad Prism software (v8.3, USA). A *p* value less than 0.05 was considered statistically significant.

## 3. Results

### 3.1. Quercetin attenuated metabolic disorders in a MetS mouse model

**Figure 1.**
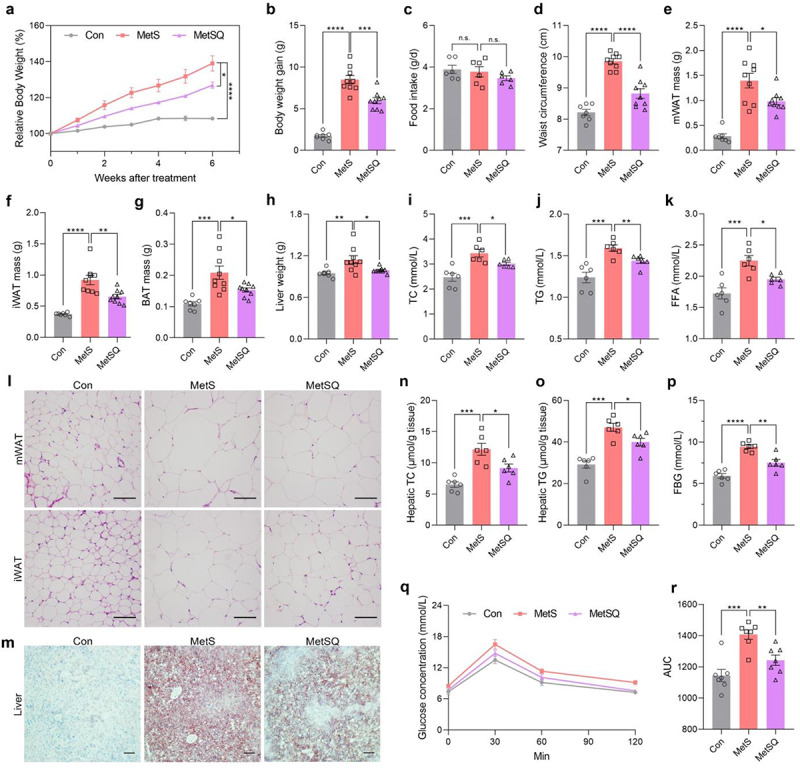
Quercetin alleviated metabolic disorders in the abdominal obesity-related metabolic syndrome (MetS) mouse model. (a) Relative body weight. (b) Body weight gain. (c) Food intake. (d) Waist circumference. (e-g) The mass of mesenteric white adipose tissues (mWAT), inguinal white adipose tissues (iWAT), and brown adipose tissues (BAT). (h) Liver weight. (i-k) Total cholesterol (TC), triglycerides (TG), and free fatty acids (FFA) concentrations in serum. (l) H&E staining images of mWAT and iWAT. Scale bar: 50 μm. (m) Oil red O staining images of liver tissues. Scale bar: 50 μm. (n, o) Hepatic TC and TG concentrations. (p) Fasting blood glucose (FBG). (q) The blood glucose concentrations in the oral glucose tolerance test (OGTT). (r) The area under curve (AUC) of the OGTT. All values are shown as mean ± s.e.m, *n*=7 in the control (Con) group, *n*=9 in the MetS group and MetSQ group in a, b, and d-h, *n*=6 per group in c, i-k and n-p, *n*=7 per group in q and r, *p < 0.05, **p < 0.01, ***p < 0.001, ****p < 0.0001.

After 6-week oral administration of quercetin at a dosage of 50 mg/kg, the MetS mice treated with quercetin exhibited significantly 29.7% lower body weight gain than the MetS mice (Figure 1(a,b)) with similar food intake (Figure 1(c)). Furthermore, the waist circumference of MetS mice was also obviously reduced by 10.5% upon quercetin treatment (Figure 1(d)). The decrease in body weight gain was partially attributed to the reduction in fat mass, including mesenteric white adipose tissue (mWAT), inguinal white adipose tissues (iWAT), and BAT, as well as liver weight, with a 29.5%, 28.9%, 26.5%, and 13.2% decrease, respectively, in the MetSQ group versus the MetS group (Figure 1(e–h)). Moreover, quercetin-treated MetS mice exhibited significantly reduced levels of TC, TG, FFA, and LDL-C in serum (Figure 1(i–k), S1), indicating alleviated lipidemia. In line with the reduction of fat weight, H&E staining results of mWAT and iWAT showed adipocytes in the MetSQ group were markedly smaller than those in the MetS group (Figure 1(l) and S2). Furthermore, analysis of liver tissues with Oil Red O staining demonstrated a reduction in lipid droplets after quercetin treatment in MetS mice (Figure 1(m)), which was also confirmed by 24.6% decrease of TC and 15.0% decrease of TG levels in liver tissues (Figure 1(n,o)). The obvious decrease in serum AST and ALT levels after quercetin treatment suggested the hepatoprotective effects of quercetin (Figure S3). Additionally, MetS mice displayed decreased FBG levels after quercetin treatment (Figure 1(p)). To further clarify the role of quercetin on the obesity-associated insulin resistance, an OGTT was performed and the results revealed improved glucose tolerance upon quercetin administration (Figure 1(q,r)). Taken together, these data revealed that oral administration of quercetin prevented metabolic disorders in MetS mice.

### 3.2. Quercetin enhanced energy expenditure

**Figure 2.**
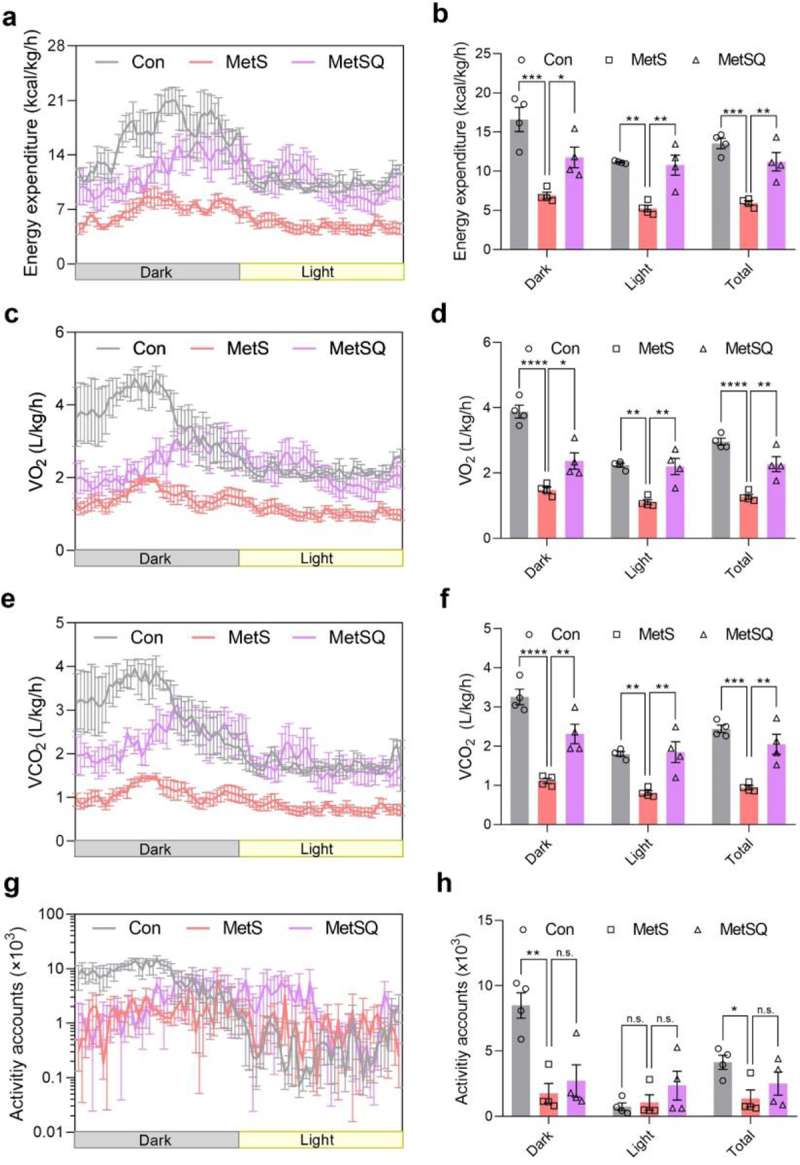
Quercetin enhanced energy metabolism in mice with MetS. (a) Energy expenditure. (b) The average energy expenditure in the 12-h dark cycle, 12-h light cycle, and total 24-h cycle. (c) VO_2_. (d) The average VO_2_ in the 12-h dark cycle, 12-h light cycle, and total 24-h cycle. (e) VCO_2_. (f) The average VCO_2_ in the 12-h dark cycle, 12-h light cycle, and total 24-h cycle. (g) Activity accounts. (h) The average activity accounts in the 12-h dark cycle, 12-h light cycle, and total 24-h cycle. Data are shown as mean ± s.e.m, *n* = 4 in each group, *p < 0.05, **p < 0.01, ***p < 0.001, ****p < 0.0001, n.s., non-significant.

Energy metabolism is closely related to obesity-related metabolic complications,^5^ therefore, the effects of quercetin on energy expenditure was evaluated. Calorimetry analyses indicated that MetSQ mice exhibited significantly higher energy expenditure than MetS mice, both during the day and at night (Figure 2(a,b)). Moreover, the 24-h overall energy expenditure of MetSQ mice was found to be 87.7% higher than that of mice in the MetS group (Figure 2(b)). Consistent with enhanced energy consumption, MetSQ mice consumed 78.5% more O_2_ and released 117.6% more CO_2_ compared to MetS mice (Figure 2(c–f)). Interestingly, no significant differences were observed in total physical activity accounts between the MetS and MetSQ groups (Figure 2(g,h)), indicating that the metabolic improvements induced by quercetin might be a result of enhanced energy expenditure rather than reduced caloric intake (Figure 1(c)) or increased physical activity (Figure 2(g,h)). These findings supported that quercetin could promote energy metabolism to deliver metabolic benefits.

### 3.3. Quercetin boosted brown adipose thermogenesis and white adipose browning

**Figure 3.**
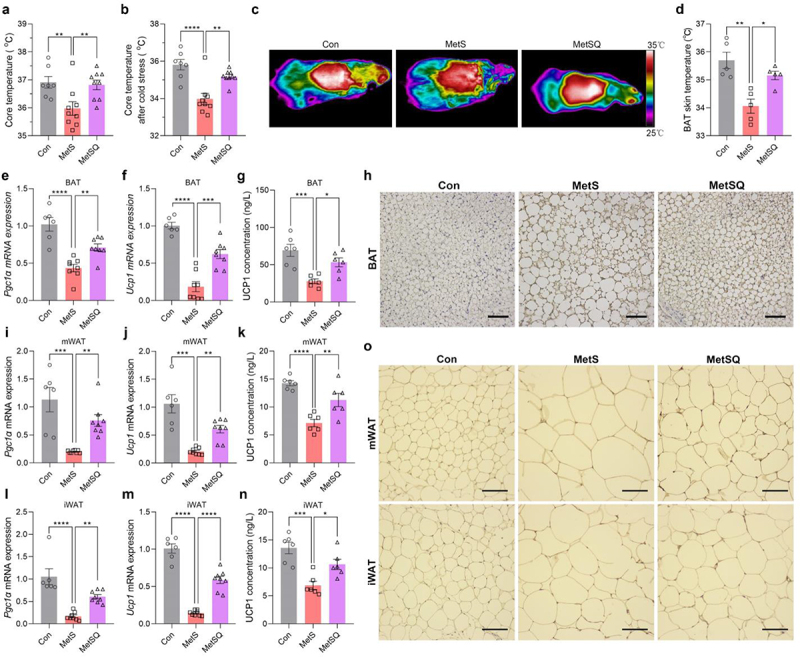
Quercetin promoted BAT thermogenesis and WAT browning in MetS mice. (a) Core temperature. (b) Core temperature after 12 h cold stress. (c) Infrared thermography of mice. (d) Interscapular BAT skin temperature. (e, f) The relative mRNA expression levels of *Pgc1α* and *Ucp1* in BAT. (g) The UCP1 concentration in BAT. (h) UCP1 immunohistochemical staining images of BAT. Scale bar: 50 μm. (i, j) The relative mRNA expression levels of *Pgc1α* and *Ucp1* in mWAT. (k) The UCP1 concentration in mWAT. (l, m) The relative mRNA expression levels of *Pgc1α* and *Ucp1* in iWAT. (n) The UCP1 concentration in iWAT. (o) UCP1 immunohistochemical staining images of mWAT and iWAT. Scale bar: 50 μm. Data are shown as mean ± s.e.m, *n*=7 in the Con group, *n*=9 in the MetS and MetSQ group in a and b, *n*=5 per group in d, *n*=6 in the Con group, *n*=8 in the MetS and MetSQ group in e, f, i, j, l, and m, *n*=6 per group in g, k, and n, *p < 0.05, **p < 0.01, ***p <0.001, ****p <0.0001.

The MetSQ mice maintained significantly higher core temperature both before and after cold exposure (Figure 3(a,b)), suggesting that quercetin treatment might enhance adaptive non-shivering thermogenesis through promoting the burning of adipose tissues. Infrared images of the mice also revealed noticeably higher BAT skin temperature in quercetin-treated MetS mice compared to those without quercetin treatment (Figure 3(c,d)), indicating the activation of BAT thermogenesis by quercetin. Moreover, the mRNA expression levels of thermogenesis-related genes, peroxisome proliferator-activated receptor γ coactivator-1 α (*Pgc1α*) and *Ucp1*, were significantly upregulated by 63.5% and 235.2% in BAT, respectively, after quercetin treatment (Figure 3(e,f)). Consistently, quercetin treatment induced substantial 91.1% more UCP1 protein expression in BAT of MetS mice, as indicated by the ELISA and immunohistochemical staining results (Figure 3(g,h)). Furthermore, the mRNA expression levels of *Pgc1α* and *Ucp1*, as well as UCP1 protein expression levels, in mWAT and iWAT were notably elevated following quercetin treatment (Figure 3(i–o)), indicating the browing of WAT. All of the data demonstrated that quercetin stimulated thermogenesis of BAT while inducing browning of WAT.

### 3.4. Quercetin elevated the production of non-12OH BAs in MetS mice

**Figure 4.**
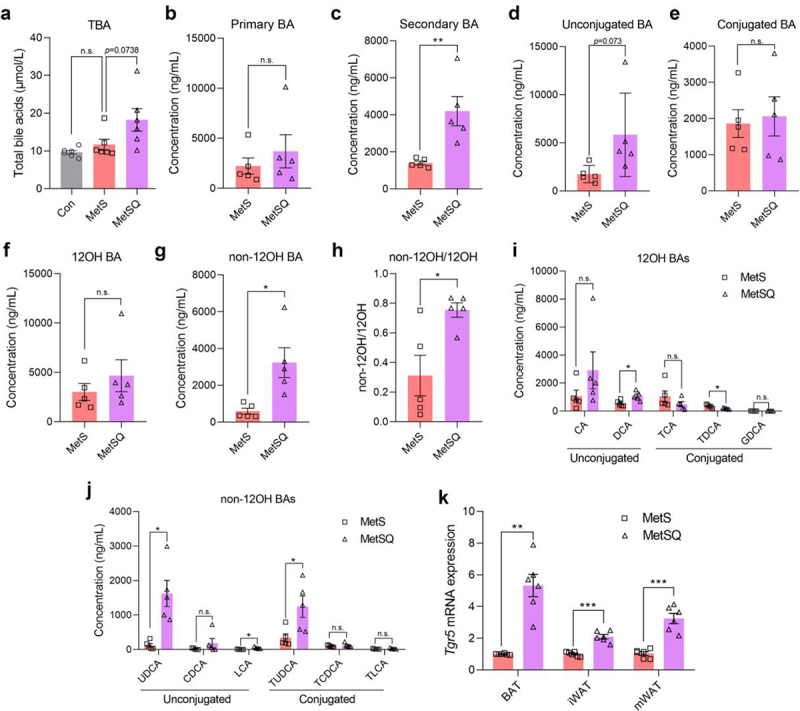
Quercetin elevated the levels of non-12OH bile acids (BAs) in sera of MetS mice. (a) Total BA concentration. (b) Primary BA concentration. (c) Secondary BA concentration. (d) Unconjugated BA concentration. (e) Conjucated BA concentration. (f) 12OH BA concentration. (g) Non-12OH BA concentration. (h) The ratio of non-12OH BA level to 12OH BA level. (i) The levels of CA, DCA, TCA, TDCA, and GDCA. (j) The levels of UDCA, CDCA, LCA, TUDCA, TCDCA, and TLCA. (k) The relative mRNA expression levels of *Tgr5* in BAT, iWAT, and mWAT. All values are shown as mean ± s.e.m, *n*=5 in a-j and *n*=6 in k in each group, *p < 0.05, **p < 0.01, ***p < 0.001, n.s., non-significant. Cholic acid (CA); deoxycholic acid (DCA); taurocholic acid (TCA); taurodeoxycholic acid (TDCA); glycodehydrocholic acid (GDCA); ursodeoxycholic acid (UDCA); chenodeoxycholic acid (CDCA); lithocholic acid (LCA); tauroursodeoxycholic acid (TUDCA); taurochenodeoxycholic acid (TCDCA); taurolithocholic acid (TLCA).

Next, we quantified the serum BA profiles in the MetS and MetSQ groups. Quercetin treatment triggered a 56.4% increase in total BA concentration in serum in MetS mice (Figure 4(a)). While no notable changes were observed in primary BA levels, there was a more than 2-fold higher concentration of secondary BAs detected in the MetSQ group (Figure 4(b,c)). The ratio of secondary BA to primary BA concentration in the MetSQ group was increased by 70.2% versus that of MetS group (Figure S4(a)). Furthermore, alterations in BA conjugation were also analyzed, the results showed quercetin induced a more pronounced elevation in unconjugated than conjugated BA concentration (Figure 4(d,e)), resulting in a significant decrease in the ratio of conjugated to unconjugated BA concentration (Figure S4(b)). More importantly, a conspicuous 4.4-fold higher concentration of non-12OH BA, but not 12α-hydroxylated BAs (12OH BA), was observed in MetSQ mice compared to MetS mice (Figure 4(f,g)). Correspondingly, the ratio of non-12OH to 12OH BA level was significantly elevated by quercetin treatment (Figure 4(h)). Concerning specific 12OH BAs, the level of deoxycholic acid (DCA) showed a significant increase, while its tauro-conjugated form, taurodeoxycholic acid (TDCA), exhibited a reduction after quercetin treatment (Figure 4(i)). More alarmingly, quercetin administration raised the concentration of non-12OH BAs, especially UDCA, LCA, and tauroursodeoxycholic acid (TUDCA), in MetS mice (Figure 4(j)). It was worth noting that UDCA and LCA levels in serum were dramatically increased by 12.5 and 7.5 times following quercetin treatment, respectively (Figure 4(j)). The binding of non-12OH BAs to Takeda G protein-coupled receptor 5 (TGR5) on adipocytes has been reported, thereby inducing the activation of browning and thermogenesis.^18,20,33^ Therefore, the *Tgr5* mRNA expression levels in adipose tissues were determined and the results showed that its expression levels in BAT, mWAT, and iWAT were much higher in the MetSQ group than those in the MetS group (Figure 4(k)), which established a link between quercetin on non-12OH BAs and energy metabolism. To further validate the relationship between UDCA and thermogenesis, the *in vitro* co-incubation of isolated brown adipocytes with UDCA was performed. It is found that 50 μM and 100 μM UDCA definitely activated the TGR5 pathway and promoted the mRNA expression of *Pgc1α* and *Ucp1* in differentiated brown adipocytes (Figure S5). Collectively, these results proved that quercetin administration elevated the production of non-12OH BAs, thereby stimulating energy metabolism in MetS mice.

### 3.5. Quercetin remodeled gut microbiota structure of MetS mice

**Figure 5.**
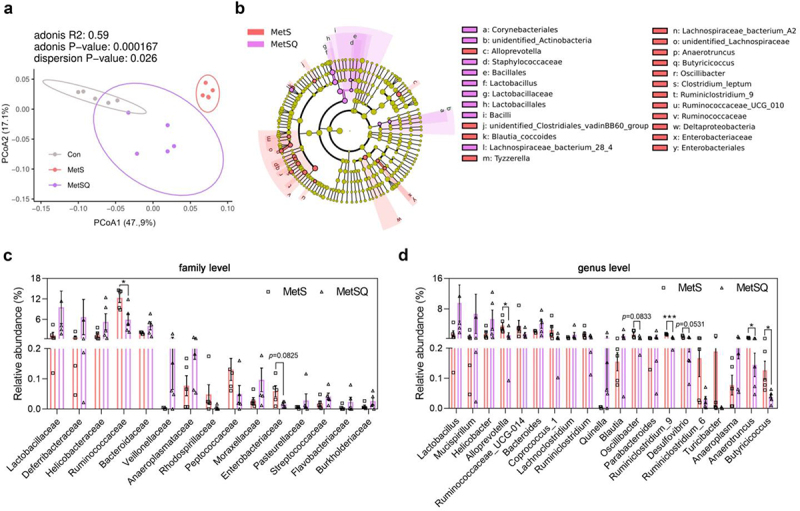
Quercetin reconstructed gut microbiota structure of MetS mice. (a) The principal coordinate analysis (PCoA) based on binary_jaccard distances. (b) The linear discriminant analysis of effect size (LEfSe) of gut microbiota between MetS and MetSQ groups. (c, d) Relative abundances of gut microbiota at family level and genus level. All values are shown as mean ± s.e.m, *n*=5 per group, *p < 0.05, ***p < 0.001.

Due to the minor effects of quercetin on the expression of BA synthesis-related genes (Figure S6), it was reasonable to suspect that another mediator of BA composition, the gut microbiota, may be involved in the regulation of BA by quercetin. To verify this hypothesis, the structure of the gut microbiota was analyzed. The α-diversity analysis revealed no significant differences in observed species, Shannon index, and Chao index among Con, MetS, and MetSQ groups (Figure S7). However, PCoA based on binary Jaccard distances and unweighted Unifrac distances displayed dramatically different clustering patterns (Figure 5(a) and S8), indicating a differential gut microbiota structure between MetSQ mice and MetS mice, which was further supported by the phylum-level analysis of gut microbiota structure (Figure S9). Then, we conducted LEfSe to further identify specific gut bacterial differences and observed that the most enriched bacteria taxa in the MetSQ group was *Lactobacillus*, with a 6.7-fold increase versus that in the MetS group (Figure 5(b), S10, S11). At the family level, the relative abundance of Ruminococcaceae in MetSQ mice was decreased by 51.7% compared to MetS mice (Figure 5(c)). Additionally, quercetin treatment led to significant reductions in absolute abundances of Ruminococcaceae, Enterobacteriaceae, and Geobacteraceae. Conversely, the absolute abundances of Staphylococcaceae and Corynebacteriaceae were considerably higher in the MetSQ group than those in the MetS group (Figure S12(a)). At the genus level, the relative abundances of *Alloprevotella*, *Ruminiclostridium_9*, *Anaerotruncus*, and *Butyricicoccus* were significantly decreased by quercetin administration (Figure 5(d)). Absolute abundance analyses showed that quercetin notably reduced *Ruminiclostridium_9*, *Desulfovibrio*, *Butyricicoccus*, *Tyzzerella*, and *Geobacter*, while enriched *Jeotgalicoccus*, *Corynebacterium_1*, and *Facklamia* in MetS mice (Figure S12(b)). These data provide evidence that quercetin reconstructed gut microbiota structure of MetS mice.

### 3.6. FMT conferred metabolic benefits of quercetin to MetS mice

**Figure 6.**
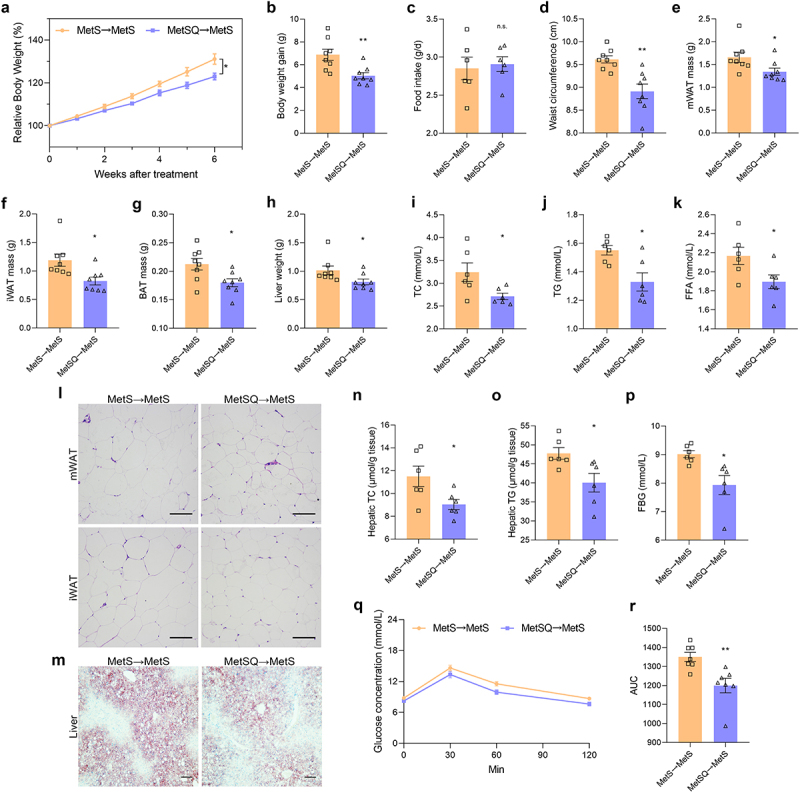
Fecal microbiota transplantation (FMT) effectively conferred metabolic benefits of quercetin to MetS mice. (a) Relative body weight. (b) Body weight gain. (c) Food intake. (d) The waist circumference. (e-g) The mass of mWAT, iWAT, and BAT. (h) Liver weight. (i-k) TC, TG, and FFA concentration in serum. (l) H&E staining images of mWAT and iWAT. Scale bar: 50 μm. (m) Oil red O staining images of liver tissues. Scale bar: 50 μm. (n, o) Hepatic TC and TG concentrations. (p) FBG. (q) The blood glucose concentration in the OGTT. (r) The AUC of the OGTT. All values are shown as mean ± s.e.m, *n*=8 per group in a, b, and d-h, *n*=6 per group in c, i-k and n-p, *n*=7 per group in q and r, *p < 0.05, **p < 0.01, n.s., non-significant.

To investigate the role of gut microbes in the metabolic benefits of quercetin, FMT was employed. We first ruled out the potential impact of quercetin in the supernatant of FMT. High-performance liquid chromatography (HPLC) analysis revealed that the concentration of quercetin in the fecal bacterial supernatant for FMT was below 10 μg/mL (Figure S13), representing a minimum of 500-fold reduction compared to the concentration used for quercetin treatment due to its low solubility in water (0.17–7 μg/mL).^34^ Following transplantation of gut microbiota from MetSQ mice, MetS mice displayed significant weight loss and reduced waist circumference without affecting food intake (Figure 6(a–d)). In line with body weight loss, there were also significant reductions in the weights of mWAT, iWAT, BAT, and liver tissues after FMT (Figure 6(e–h)). The remarkable decrease of TC, TG, LDL-C, and FFA concentrations in serum indicated that FMT effectively delivered the beneficial effects of quercetin on lipidemia to MetS mice (Figure 6(i–j), S14). Furthermore, adipocytes in MetSQ→MetS mice were significantly smaller than those in MetS→MetS mice (Figure 6(l), S15). Additionally, MetSQ→MetS mice exhibited significantly reduced hepatic fat deposition (Figure 6(m–o), S16). The significant decrease of FBG levels and improvement in OGTT suggested that FMT exerted ameliorative effects on obesity-related insulin resistance (Figure 6(p–r)). All of the data suggested FMT conferred metabolic benefits of quercetin to MetS mice.

### 3.7. Reduced energy expenditure was improved by FMT in MetS mice

**Figure 7.**
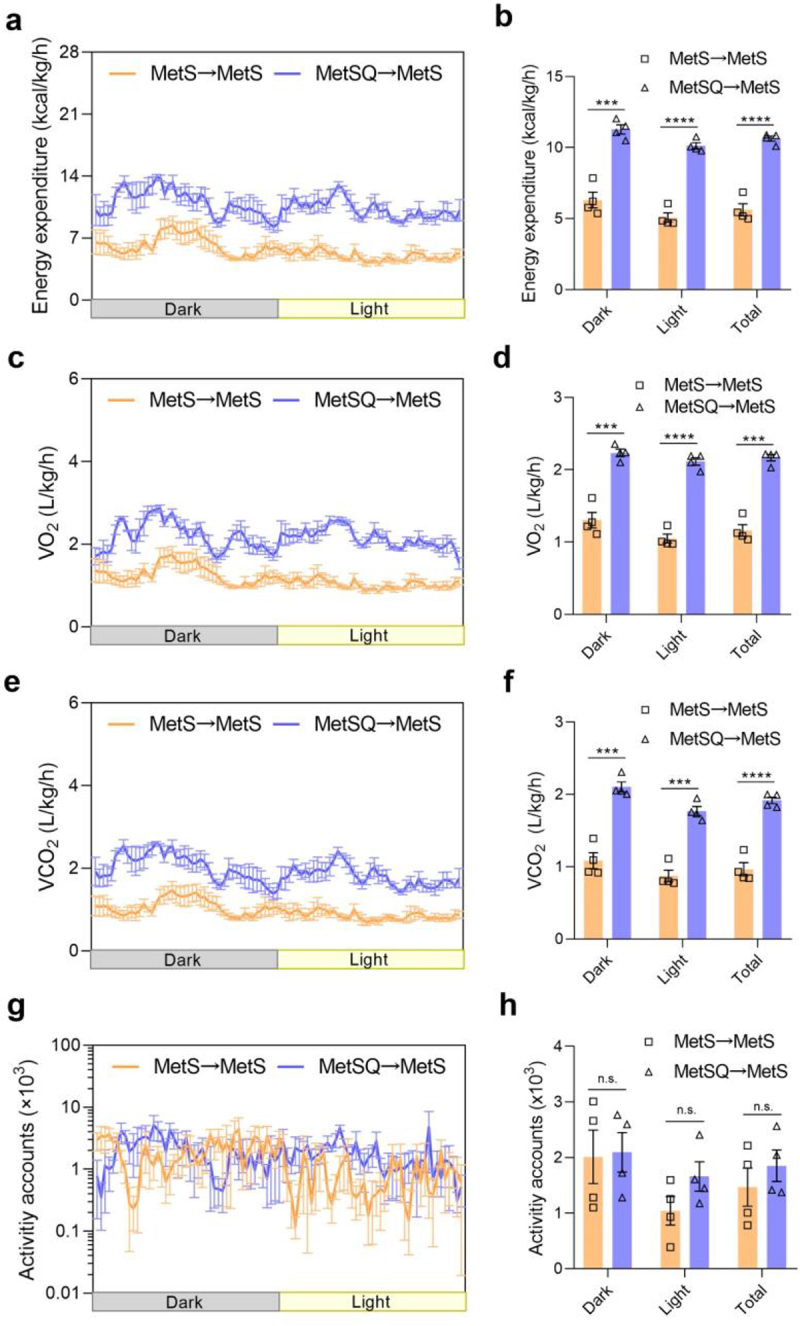
FMT promoted energy expenditure. (a) Energy expenditure. (b) The average energy expenditure in the 12-h dark cycle, 12-h light cycle, and total 24-h cycle. (c) VO_2_. (d) The average VO_2_ in the 12-h dark cycle, 12-h light cycle, and total 24-h cycle. (e) VCO_2_. (f) The average VCO_2_ in the 12-h dark cycle, 12-h light cycle, and total 24-h cycle. (g) Activity accounts. (h) The average activity counts in the 12-h dark cycle, 12-h light cycle, and total 24-h cycle. Data are shown as mean ± s.e.m, *n*=4 per group, ***p<0.001, ****p<0.0001, n.s., non-significant.

Next, we explored the potential benefits of FMT on energy metabolism in MetS mice. The metabolic analyses showed that MetSQ→MetS mice displayed a remarkable 89.3% increase in energy expenditure compared to MetS→MetS mice over a 24 h period (Figure 7(a,b)). Consistent with the elevated energy expenditure, MetSQ→MetS mice consumed an additional 84.6% of O_2_ and produced an extra 96.7% of CO_2_ compared to MetS→MetS mice (Figure 7(c–f)). Moreover, there was no significant difference in total physical activity accounts between the two groups of FMT-recipient mice (Figure 7(g,h)). These results highlighted that transplanting gut microbiota from quercetin-treated mice ameliorated energy metabolism disorders in MetS mice.

### 3.8. FMT stimulated BAT thermogenesis and WAT browning

**Figure 8.**
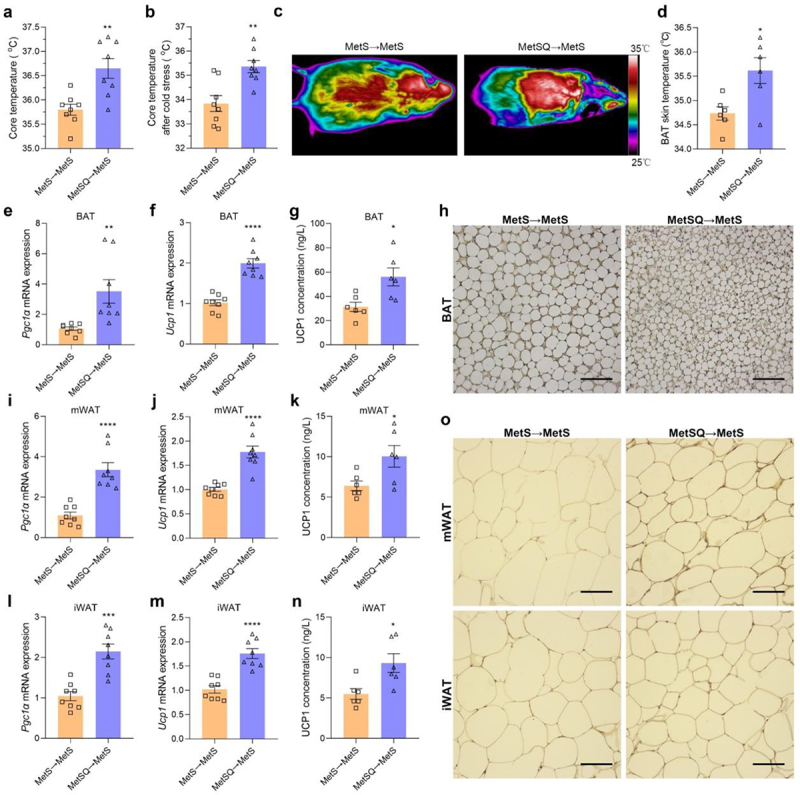
FMT enhanced thermogenesis of BAT and browning of WAT in MetS mice. (a) Core temperature. (b) Core temperature after 12 h cold stress. (c) Infrared thermography of mice. (d) Interscapular BAT skin temperature. (e, f) The relative mRNA expression levels of *Pgc1α* and *Ucp1* in BAT. (g) The UCP1 concentration in BAT. (h) UCP1 immunohistochemical staining images of BAT. Scale bar: 50 μm. (i, j) The relative mRNA expression levels of *Pgc1α* and *Ucp1* in mWAT. (k) The UCP1 concentration in mWAT. (l, m) The relative mRNA expression levels of *Pgc1α* and *Ucp1* in iWAT. (n) The UCP1 concentration in iWAT. (o) UCP1 immunohistochemical staining images of mWAT and iWAT. Scale bar: 50 μm. Data are shown as mean ± s.e.m, *n*=8 per group in a and b, *n*=6 per group in d, g, k, and n, *n*=8 in per group in e, f, i, j, l, and m, *p< 0.05, **p< 0.01, ***p<0.001, ****p<0.0001.

The effects of FMT on thermogenesis of BAT and browning of WAT was evaluated. As expected, MetSQ→MetS mice were more resistant to core temperature loss upon cold challenge than MetS→MetS mice (Figure 8(a,b)). The higher BAT skin temperature as indicated by infrared images also proved this conclusion (Figure 8(c,d)). Additionally, MetSQ→MetS mice showed noticeable increases in *Pgc1α* and *Ucp1* mRNA and UCP1 protein levels in BAT, as well as in mWAT and iWAT (Figure 8(e–o)). Hence, the results supported the conclusion that the stimulative effects of quercetin on BAT thermogenesis and WAT browning were gut microbiota-dependent.

### 3.9. The levels of non-12OH BAs were increased after FMT

**Figure 9.**
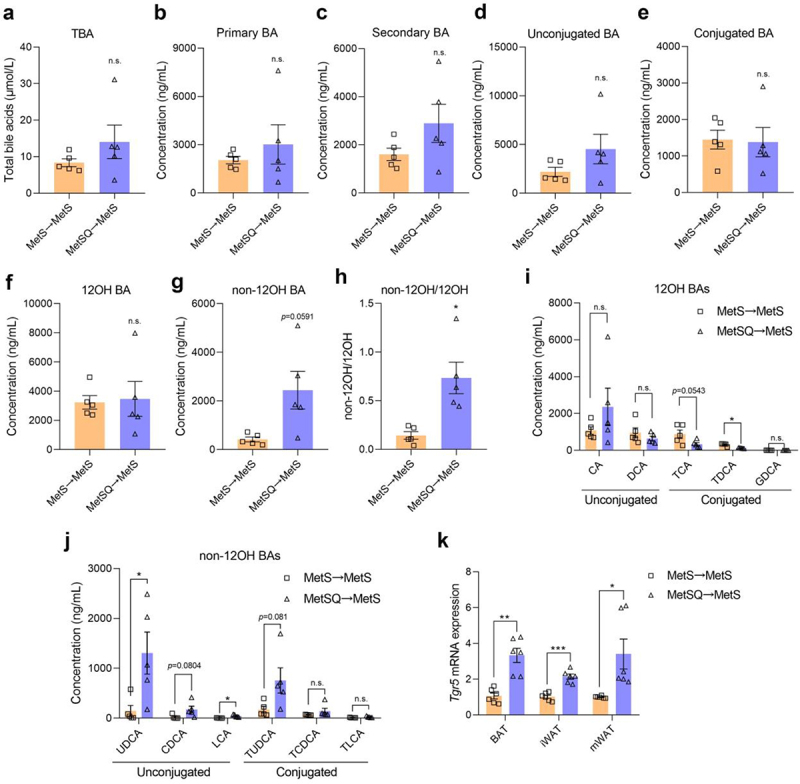
FMT increased non-12OH BA production. (a) Total BA concentration. (b) Primary BA concentration. (c) Secondary BA concentration. (d) Unconjugated BA concentration. (e) Conjucated BA concentration. (f) 12OH BA concentration. (g) Non-12OH BA concentration. (h) The ratio of non-12OH BA to 12OH BA level. (i) The levels of CA, DCA, TCA, TDCA, and GDCA. (j) The levels of UDCA, CDCA, LCA, TUDCA, TCDCA, and TLCA. (k) The relative mRNA expression levels of *Tgr5* in BAT, iWAT, and mWAT. All values are shown as mean ± s.e.m, *n*=5 in a-j and *n*=6 in k per group, *p< 0.05, **p< 0.01, ***p<0.001, n.s., non-significant. Cholic acid (CA); deoxycholic acid (DCA); taurocholic acid (TCA); taurodeoxycholic acid (TDCA); glycodehydrocholic acid (GDCA); ursodeoxycholic acid (UDCA); chenodeoxycholic acid (CDCA); lithocholic acid (LCA); tauroursodeoxycholic acid (TUDCA); taurochenodeoxycholic acid (TCDCA); taurolithocholic acid (TLCA).

BA composition in serum was also determined after FMT. The results showed no significant changes in the concentrations of TBA, primary BA, secondary BA, unconjugated BA, and conjugated BA between MetSQ→MetS and MetS→MetS groups (Figure 9(a–e), S17). Compared to the slight change in 12OH BA level, the non-12OH BA level in MetSQ→MetS mice was 4.8-fold higher than that in MetS→MetS mice (Figure 9(f,g)). Consistently, the ratio of non-12OH to 12OH BA concentration was significantly elevated by 413.9% after receiving FMT from quercetin-treated MetS mice (Figure 9(h)). Among 12OH BAs, TCA and TDCA levels were reduced in the MetSQ→MetS group (Figure 9(i)). More importantly, MetSQ→MetS mice exhibited obviously increased UDCA and LCA levels compared to MetS→MetS mice by 9.0 and 20.5 times (Figure 9(j)). Moreover, the mRNA expression of *Tgr5* in BAT, iWAT, and mWAT were significantly induced in response to the elevated levels of non-12OH BA in MetSQ→MetS mice (Figure 9(k)). Moreover, the abundance of *Lactobacillus* was found to be 5.0 times higher in MetSQ→MetS group than that in MetS→MetS group (Figure S18). These data suggested that FMT from MetSQ mice did replicate the stimulative effects of quercetin on non-12OH BAs production.

## 4. Discussion

The metabolic benefits of quercetin have been reported in animal and human studies.^21^ However, the underlying mechanism has not been fully investigated. In this study, we provided compelling evidence that gut microbiota-mediated secondary non-12OH BAs production significantly contributed to the combatting effects of quercetin on abdominal obesity-related MetS via promoting BAT thermogenesis and WAT browning to boost energy expenditure. Furthermore, FMT replicated quercetin’s enhancing effects on non-12OH BAs generation and energy expenditure, highlighting the decisive role of gut microbiota reshaping by quercetin in the process. Therefore, this study not only provided valuable insights into the causal links among gut microbes, non-12OH BAs, and energy expenditure in ameliorating abdominal obesity-related MetS by quercetin, but also demonstrated the potential of flavonoids in regulating gut microbiota-BAs crosstalk for preventing MetS and related diseases.

Abdominal obesity is considered a primary initiating factor in the pathogenesis of MetS.^35,36^ It is characterized by excessive accumulation of abdominal fat, distinct from general obesity with overall-body distribution of body fat.^27^ Extensive research conducted over the past three decades has consistently demonstrated that abdominal fat deposition plays a pivotal role in contributing to, and potentially driving, the associated health risks of MetS.^27,37^ The detrimental impact of abdominal obesity on diseases such as diabetes and cardiovascular diseases surpasses that of general obesity.^36,37^ Therefore, the monosodium glutamate-induced MetS mouse model characterized by abdominal obesity was selected as the research object in this study. The ameliorative effects of quercetin on abdominal obesity not only extend previous studies utilizing diet-induced obesity animal models but also deepen our understanding regarding the mechanism underlying quercetin’s benefits on metabolic diseases through gut microbiota-BAs crosstalk.

Brown and beige adipose tissues, in contrast to WAT which primarily stores energy, controls energy expenditure, so activating BAT and promoting WAT browning are considered effective therapeutic strategies for metabolic diseases.^4^ UCP1-positive adipocytes play a crucial role in thermogenesis by generating heat through dissipation of the proton gradient produced by the electron transport chain.^38^ UCP1 induction by various stimuli including cold, exercise, and diets enhances nonshivering thermogenesis, leading to increased energy expenditure and prevention of obesity-associated metabolic diseases.^8,39^ However, adult mammals, especially humans, have limited BAT mass and activity, as well as beige adipose tissues. The amount of BAT in an adult’s body ranges 50–500 g, and the presence of BAT was independently correlated with lower odds of type 2 diabetes and dyslipidemia.^40,41^ Mirabegron, a β3-adrenergic receptor agonist, has been proven to significantly activate the activity of BAT in healthy individuals, resulting in 13% more energy expenditure.^42,43^ More importantly, studies investigating the influence of environmental temperature on BAT content suggest that human BAT is malleable.^44^ These findings underscore the potential and necessity for identifying effective drugs and technologies to activate BAT in preclinical studies and achieve clinical translation. Our data suggested quercetin could induce the expression and activation of UCP1 in both BAT and WAT to generate metabolic improvements in MetS mice, which implied that quercetin might be a promising drug to activate human BAT against metabolic disorders, but further clinical validation was required. Further results of FMT in this study supported that microbial regulation of quercetin contributed to the upregulation of UCP1 in adipose tissues. It was also found that both quercetin treatment and colonization with the microbiota from quercetin-treated mice enriched *Lactobacillus* and non-12OH BAs, indicating that UCP1 in adipose tissues might serve as an important downstream effector of *Lactobacillus* and non-12OH BAs. Nevertheless, the molecular mechanism by which gut microbes affect UCP1 expression remains to be further studied.

Quercetin belongs to the broad category of polyphenolic compounds, predominantly existing as glycosides and aglycones in plants.^21^ The daily dietary intake of quercetin by human ranges from 10 to 500 mg.^45^ This study referred to a daily dose of 50 mg/kg used in previous animal studies, which is equivalent to a human dose of 284.6 mg per day for a person weighing 70 kg based on the mouse-human dose conversion.^45,46^ In addition, doses up to 2000 mg quercetin per day showed mild or no symptoms of overdose in human.^47^ Further studies are required to decide optimal dosage of quercetin for its biological effects in different diseases. Although most dietary intake of quercetin by humans comes from its derivatives, which may differ from oral absorption and bioavailability compared to pure quercetin, this does not impede the application of quercetin as a drug or food supplement for clinical trials targeting metabolic diseases due to its beneficial effects.^48^ Traditional pharmacological studies believe that the anti-obesity effect of quercetin is attributed to its excellent direct anti-inflammatory and adipogenesis-suppressed properties.^49,50^ However, the relatively low oral absorption and bioavailability of quercetin and its form of glycosides limit these effect in distal target tissues, such as liver and adipose tissues.^34,51^ As the primary site of quercetin exposure, gut microbes rapidly respond upon contact with quercetin. There are evidences suggesting a connection between gut bacteria and metabolic improvements induced by quercetin.^52^ Zhao *et al*. investigated the anti-obesity effects of quercetin in an abdominal obesity mouse model, and they found quercetin administration enriched the Bacteroides population and reversed the Fimicutes/Bacteroides ratio.^53^ Etxeberria *et al*. found quercetin supplementation posed a great impact on gut bacterial composition at various taxonomic levels, including correcting Firmicutes/Bacteroidetes ratio and inhibiting the growth of bacterial species associated with obesity (Erysipelotrichaceae, Bacillus, and *Eubacterium cylindroides*) in high-fat high-sucrose diet-fed rats.^54^ Other experiments showed combined administration of *Akkermansia muciniphila* and quercetin obviously altered the gut microbiota composition, thus ameliorating obesity and non-alcoholic fatty liver disease (NAFLD).^55^ Here, we reported that quercetin treatment significantly enriched *Lactobacillus* belonging to Lactobacillaceae family within Firmicutes phylum, while reducing the abundances of OTUs in Ruminococcaceae family. It is worth noting that *Lactobacillus* could convert primary BAs into secondary BAs, such as LCA, DCA, and UDCA.^56–58^ Dietary *Lactobacillus reuteri* has been demonstrated to modify the constituents of BA pool, particularly by increasing the UDCA level, thus providing protection against liver injury.^59^ Similarly, another bacterial species within *Lactobacillus* genus, *Lactiplantibacillus plantarum H-87*, was also capable to elevate UDCA to prevent HFD-induced obesity.^60^ These findings align with the results obtained in this study, which revealed enhanced production of UDCA following *Lactobacillus* enrichment by quercetin administration.

A notable finding of this reseach was the significant increase of non-12OH BAs in BA pool in response to quercetin administration. Primary 12OH and non-12OH BAs are synthesized from cholesterol in the liver by two various pathways, the classic pathway and the alternative pathway, respectively. The classical pathway is initiated by cholesterol 7α-hydroxylase (CYP7A1) to produce chenodeoxycholic acid (CDCA) or cholic acid (CA), and the alternative pathway is initiated mainly by sterol 27-hydroxylase (CYP27A1) to generate CDCA.^61^ Primary BAs produced in liver are metabolized by gut microbes to form secondary BAs, for example, CDCA can be converted to UDCA and LCA within certain species from *Lactobacillus* and other gut bacteria.^56–58^ Although the BA profiles of human and mouse are different, UDCA and LCA are common BAs of both, which indicates the clinical translation prospect of this study.^61^ UDCA is the primary drug approved by the US Food and Drug Administration (FDA) for the treatment of biliary cirrhosis, while animal studies in recent years have shown its metabolic benefits.^20,62^ A mixture of UDCA and LCA was shown to improve lipid metabolism disorders in ob/ob obese mice.^63^ And sole UDCA supplementation also attenuated weight rebound after a restricted diet by activating thermogenesis in mice fed a HFD.^16^ Non-12OH BAs, including CDCA, LCA and UDCA, are potential TGR5 agonists to stimulate UCP1-dependent energy expenditure.^20,33^ Here, we saw *Tgr5* and *Ucp1* mRNA expression in WAT and BAT was strongly enhanced after quercetin administration, which confirmed the role of non-12OH BAs on thermogenic effects of quercetin. The biological effects of non-12OH BAs are diverse, such as their effects on intestinal barrier function and FXR receptors,^20^ whether these effects regulate quercetin’s metabolic benefits requires further investigation. It is worth mentioning that quercetin treatment did not affect the expression of genes involved in BAs synthesis, in line with the results of CA and CDCA levels without significant differences, which also indirectly proved that the increase in the non-12OH/12OH BAs ratio was due to the regulation of intestinal microbiota by quercetin.

Nevertheless, there are several limitations of this study. First, the effects of quercetin under thermal neutral (29 ℃) conditions with no intrinsic UCP1 activation was not investigated due to limitations in the experimental environment and equipment, which requires further research. Second, we did not detect other gut microbiota-derived metabolites, such as short-chain fatty acids (SCFAs), which have also been shown to affect BAT activity. Thirdly, the human gut flora structure and bile acid profile are different from those of rodents, so the conclusions in this study need to be further confirmed in clinical studies.

## 5. Conclusion

**Figure 10.**
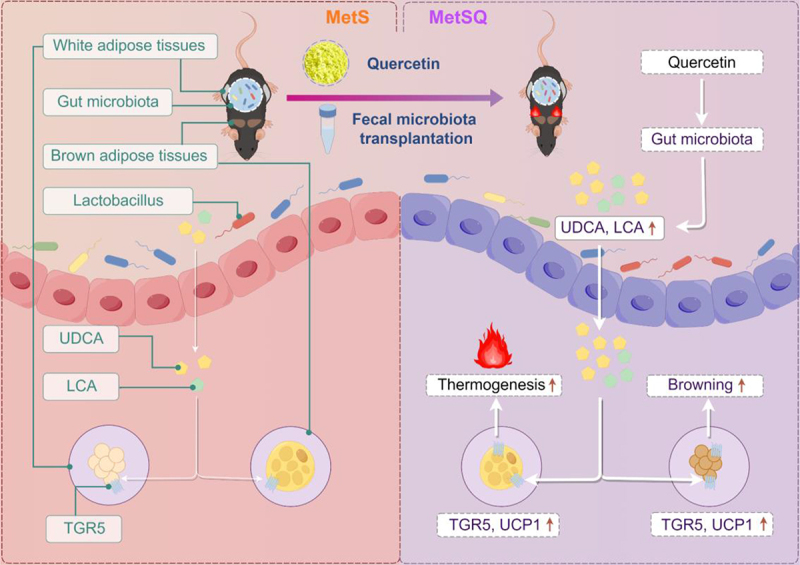
The schematic diagram showing that quercetin enhanced the production of non-12OH BAs, especially UDCA and LCA, through modulating the overall structure of gut microbiota and enriching *Lactobacillus*, thereby promoting BAT thermogenesis and WAT browning to ameliorate metabolic disorders.

Overall, our results demonstrated that quercetin enhanced the production of non-12OH BAs, especially UDCA and LCA, through modulating the structure of gut microbiota and enriching *Lactobacillus*, thereby facilitating BAT thermogenesis and WAT browning to combat abdominal obesity-related metabolic disorders (Figure 10). Our findings delineated the pivotal role of gut microbiota-BAs crosstalk in the anti-MetS property of quercetin, which enriched the pharmacological mechanisms of quercetin in the treatment of obesity-related diseases. Furthermore, our research here also highlighted the potential of developing quercetin-based nutritional supplements for preventing abdominal obesity-related MetS.

## Supplementary Material

Supplemental Material

## Data Availability

The 16S rDNA gene amplicon sequencing data used in this study have been deposited in the Genome Sequence Archive (GSA) in National Genomics Data Center (accession number: CRA016321) that are publicly accessible at http://ngdc.cncb.ac.cn/gsa. Other data supporting this research are available from the corresponding authors on reasonable request.
