# Correction

**DOI:** 10.1080/19490976.2025.2471120

**Published:** 2025-02-23

**Article title**: Quercetin activates energy expenditure to combat metabolic syndrome through modulating gut microbiota-bile acids crosstalk in mice

**Authors**: Zhu, X., Dai, X., Zhao, L., Li, J., Zhu, Y., He, W., … Lei, L.

**Journal:** *Gut Microbes*

**DOI:** https://doi.org/10.1080/19490976.2024.2390136

Due to the authors’ oversight, the Figure 6m was incorrect in the published version which is now corrected and republished. The updated figure 6 is provided below.

**Figure 6.**
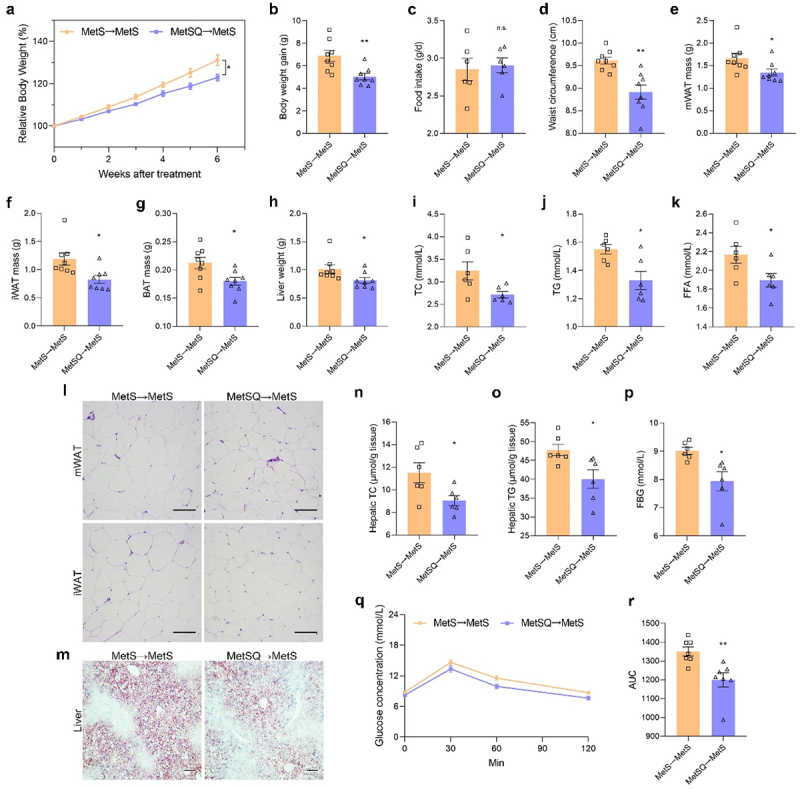
Fecal microbiota transplantation (FMT) effectively conferred metabolic benefits of quercetin to MetS mice. (a) Relative body weight. (b) Body weight gain. (c) Food intake. (d) The waist circumference. (e-g) The mass of mWAT, iWAT, and BAT. (h) Liver weight. (i-k) TC, TG, and FFA concentration in serum. (l) H&E staining images of mWAT and iWAT. Scale bar: 50 μm. (m) Oil red O staining images of liver tissues. Scale bar: 50 μm. (n, o) Hepatic TC and TG concentrations. (p) FBG. (q) The blood glucose concentration in the OGTT. (r) The AUC of the OGTT. All values are shown as mean ± s.e.m, *n*=8 per group in a, b, and d-h, *n*=6 per group in c, i-k and n-p, *n*=7 per group in q and r, *p < 0.05, **p < 0.01, n.s., non-significant.

