# Supplementary material for: Quercetin activates energy expenditure to combat metabolic syndrome through modulating gut microbiota-bile acids crosstalk in mice
Source: Gut Microbes. 2024 Aug 20;16(1):2390136. doi: 10.1080/19490976.2024.2390136 (PMC11340765; doi:10.1080/19490976.2024.2390136)
Supplement: Supplemental Material [file KGMI_A_2390136_SM0713.zip › Supplementary_Material clean.docx]

Supplementary Material

**Quercetin activates energy expenditure to combat metabolic syndrome through modulating gut microbiota-bile acids crosstalk in mice**

**Xiaoqiang Zhu**^a,b,d#*^**, Xiaojuan Dai**^c#^**, Lijun Zhao**^d,e#^**, Jing Li**^f^**, Yanhong Zhu**^d^**, Wenjuan He**^a,b^**, Xinlei Guan**^a,b^**, Tao Wu**^a,b^**, Li Liu**^b^**, Hongping Song**^b^**, Liang Lei**^a,b*^

^a^ Central Laboratory, Wuhan Fourth Hospital, Wuhan, China

^b^ Department of Pharmacy, Wuhan Fourth Hospital, Wuhan, China

^c^ Department of Gastroenterology, Wuhan Fourth Hospital, Wuhan, China

^d^ National Engineering Research Center for Nanomedicine, College of Life Science and Technology, Huazhong University of Science and Technology, Wuhan, China

^e^ Hubei Jiangxia Laboratory, Wuhan, China

^f^ Pharmaceutical Department, Hubei Cancer Hospital, Tongji Medical College, Huazhong University of Science and Technology, Wuhan, China.

^#^ Xiaoqiang Zhu, Xiaojuan Dai, and Lijun Zhao contributed equally to this work.

^*^ Correspondence: Xiaoqiang Zhu, zhuxiaoqiang1992@126.com; Liang Lei, leiliang@hust.edu.cn


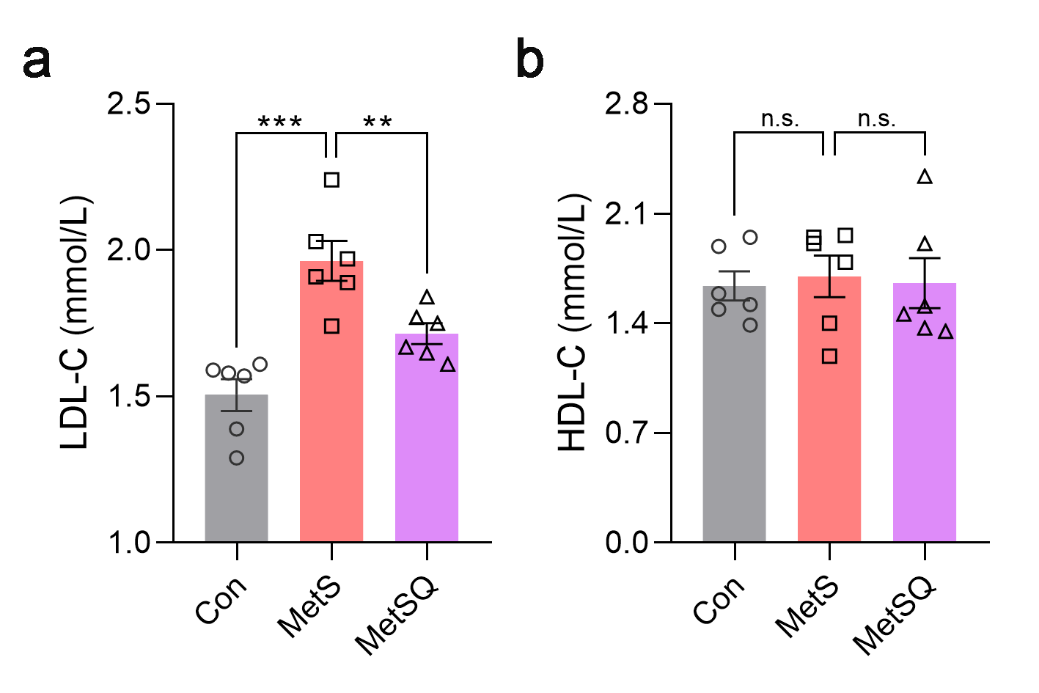


Figure S1. The (a) LDL-C and (b) HDL-C levels in serum of Con, MetS, and MetSQ groups. Related to Figure 1. All values are shown as mean ± s.e.m, n=6 in each group, ***p*<0.01, ****p*<0.001, n.s., non-significant.


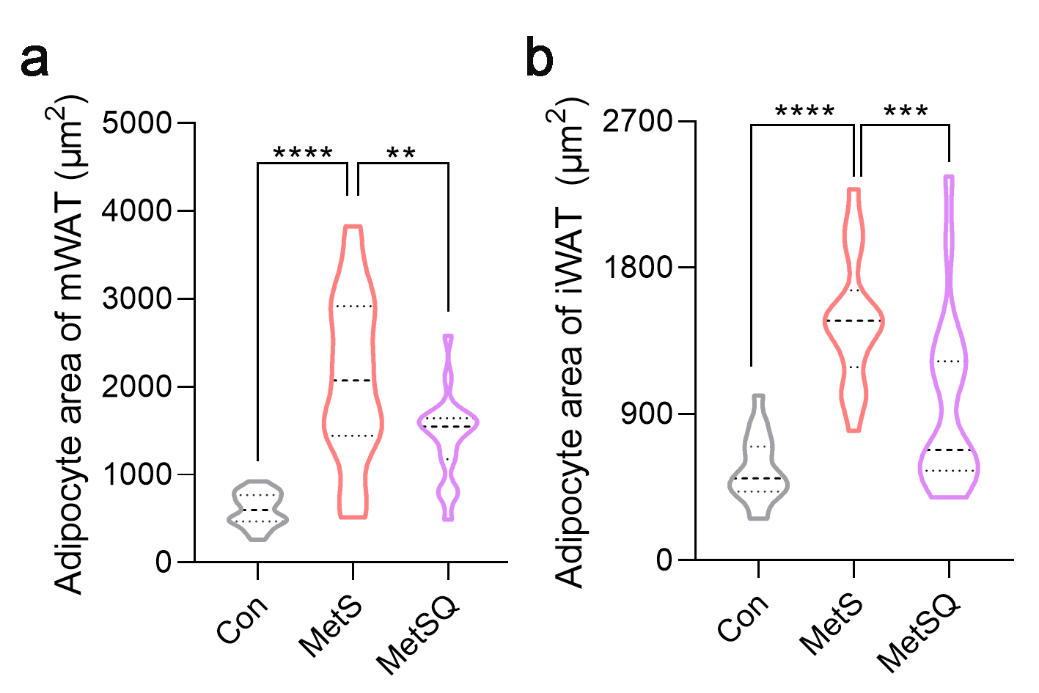


Figure S2. The average adipocyte area of (a) mWAT and (b) iWAT. Related to Figure 1. All values are shown as mean ± s.e.m, n=18-20 in each group, ***p*< 0.01, ****p*< 0.001, *****p*< 0.0001.


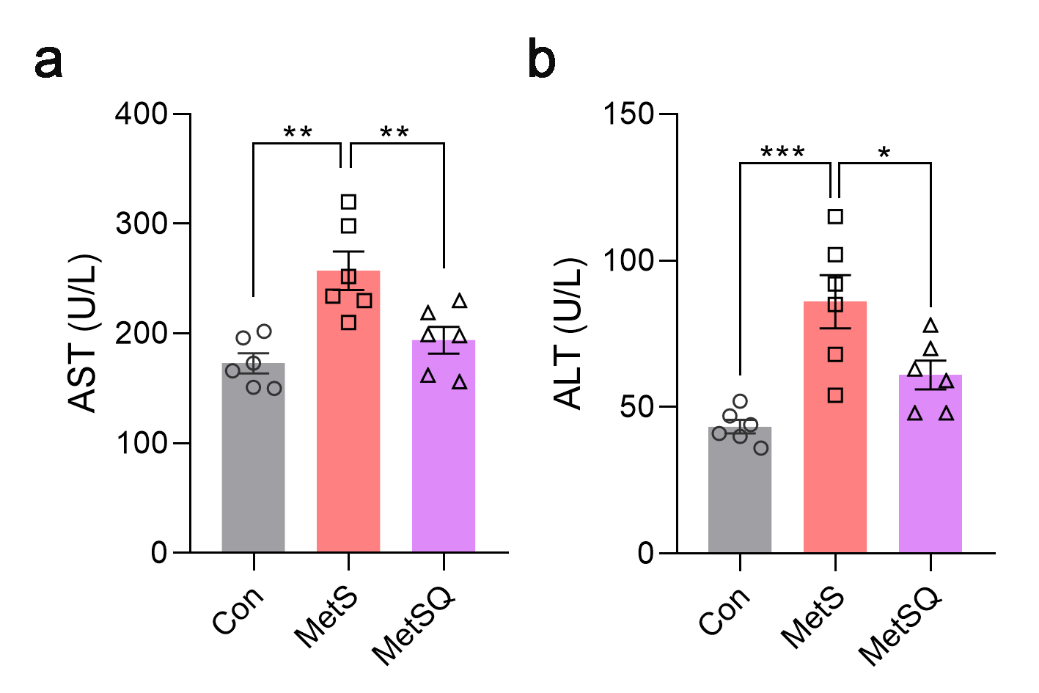


Figure S3. The (a) AST and (b) ALT levels in serum of Con, MetS, and MetSQ groups. Related to Figure 1. All values are shown as mean ± s.e.m, n=6 in each group, **p*< 0.05, ***p*< 0.01, ****p*<0.001.


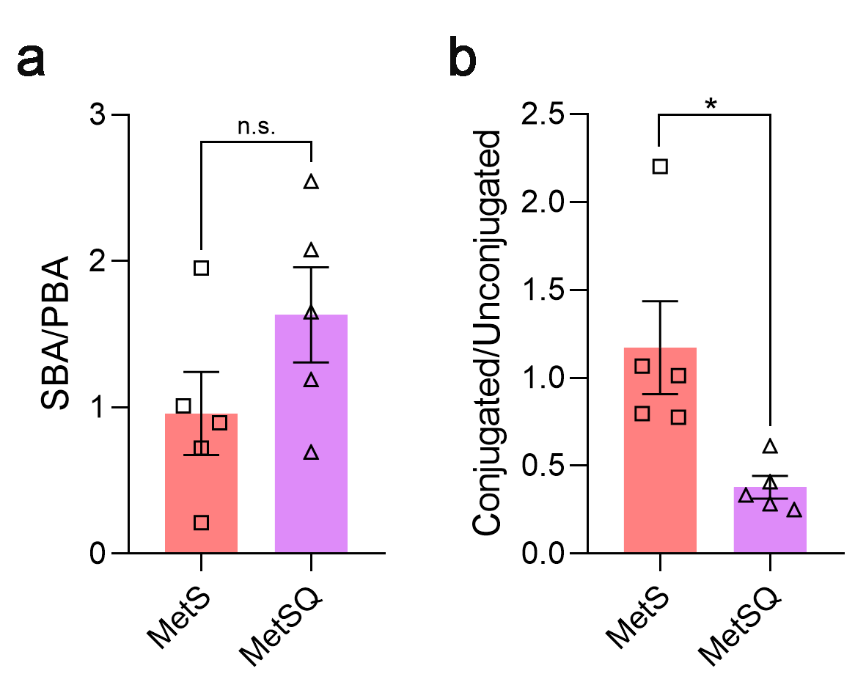


Figure S4. The ratios of (a) secondary BA (SBA) concentration to primary BA (PBA) concentration and (b) conjugated BA concentration to unconjugated BA concentration. Related to Figure 4. All values are shown as mean ± s.e.m, n=5 in each group, **p*< 0.05, *****p*<0.0001.


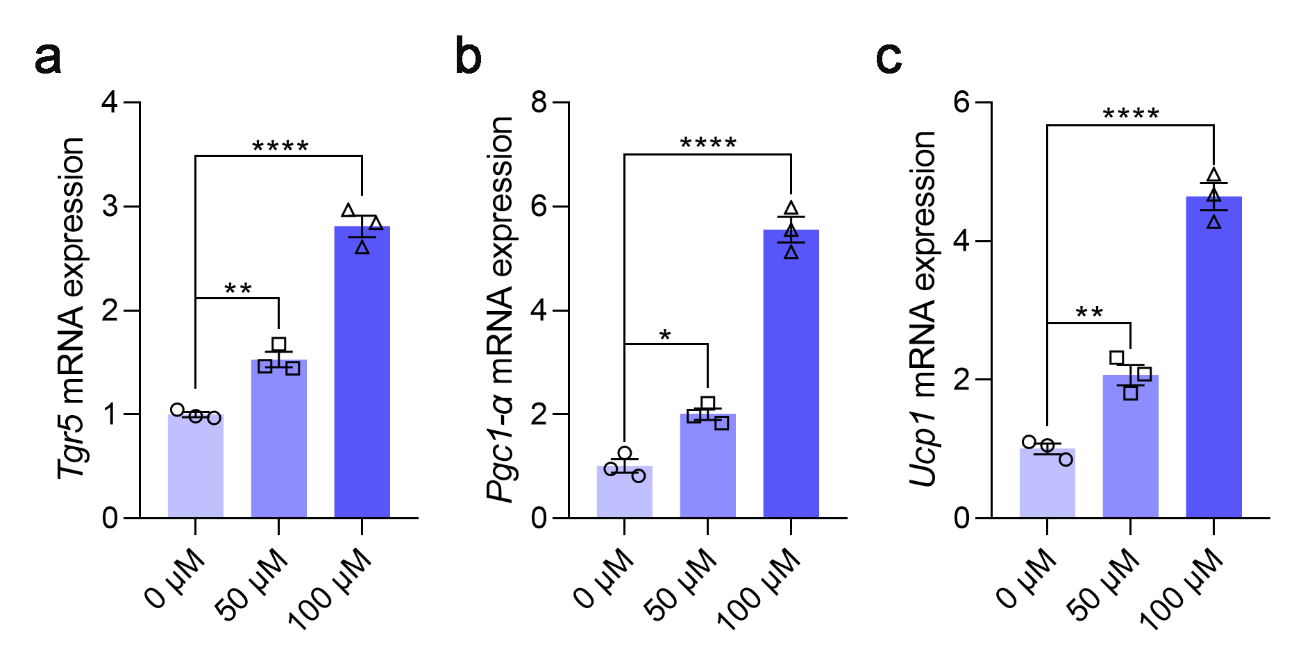


Figure S5. The relative mRNA expression levels of (a) *Tgr5*, (b) *Pgc1α*, and (c) *Ucp1* in brown adipocytes treated with different concentrations of UDCA*.* Related to Figure 4. All values are represented as mean ± s.e.m, n=3 in each group. **p*< 0.05, ***p*< 0.01, *****p*< 0.0001.


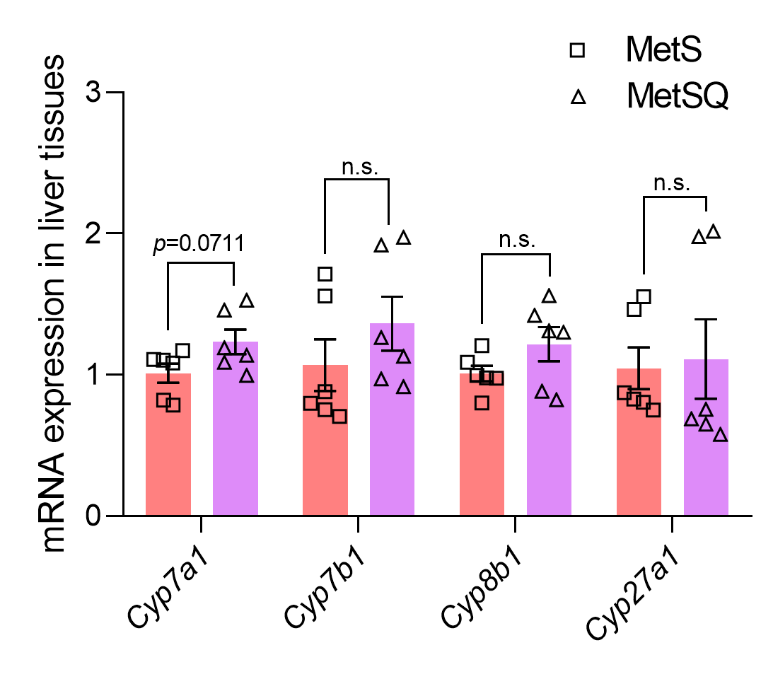


Figure S6. The relative mRNA expression levels of genes involved in BA synthesis in liver tissues. Related to Figure 4. All values are shown as mean ± s.e.m, n=6 in each group, ***p*< 0.01, ****p*<0.001.


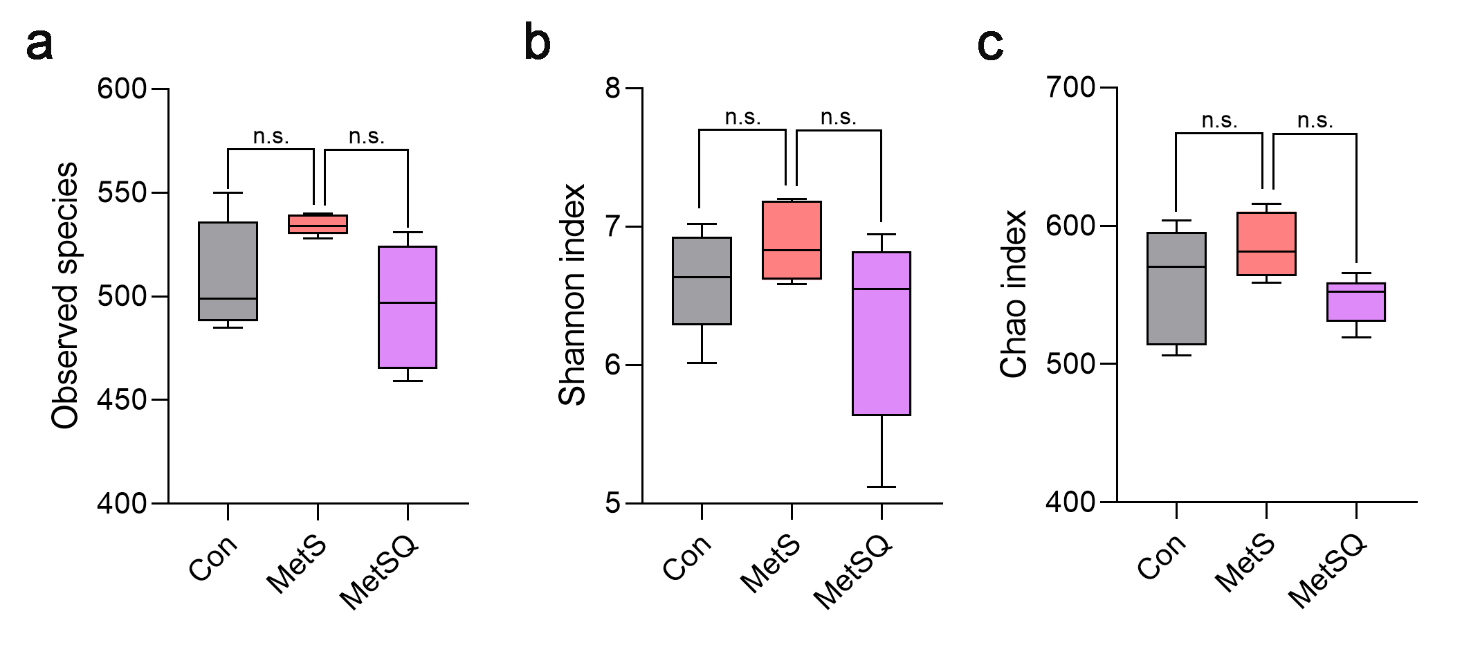


Figure S7. The α diversity of gut microbiota. Related to Figure 5. (A) Observed species. (B) Shannon index. (C) Chao index. Data are shown as interquartile range, n=5 in each group, **p*< 0.05, n.s., non-significant.


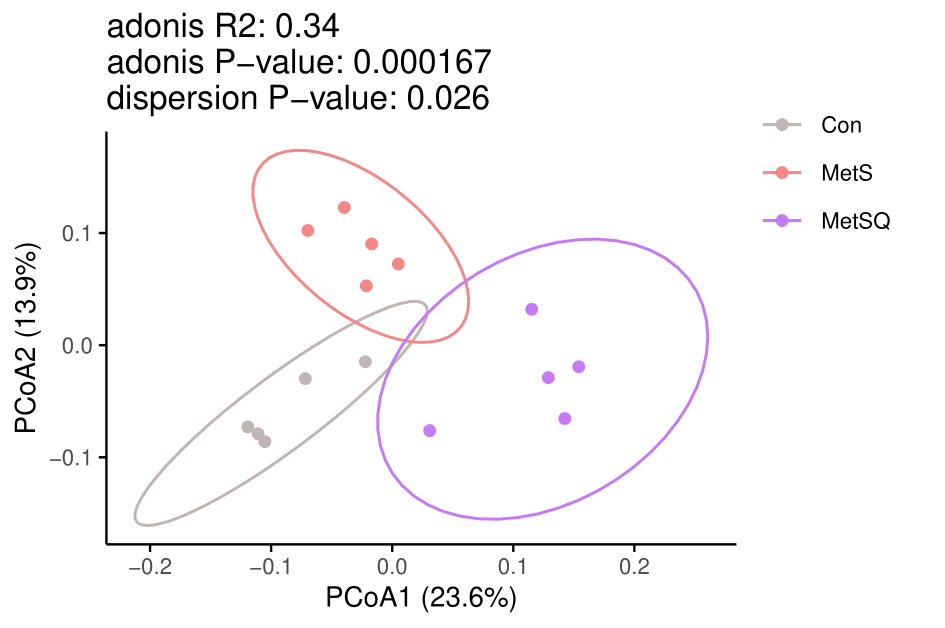


Figure S8. PCoA of gut microbiota among Con, MetS, and MetSQ groups based on Unweighted Unifrac distances. Related to Figure 5. n=5 in each group.


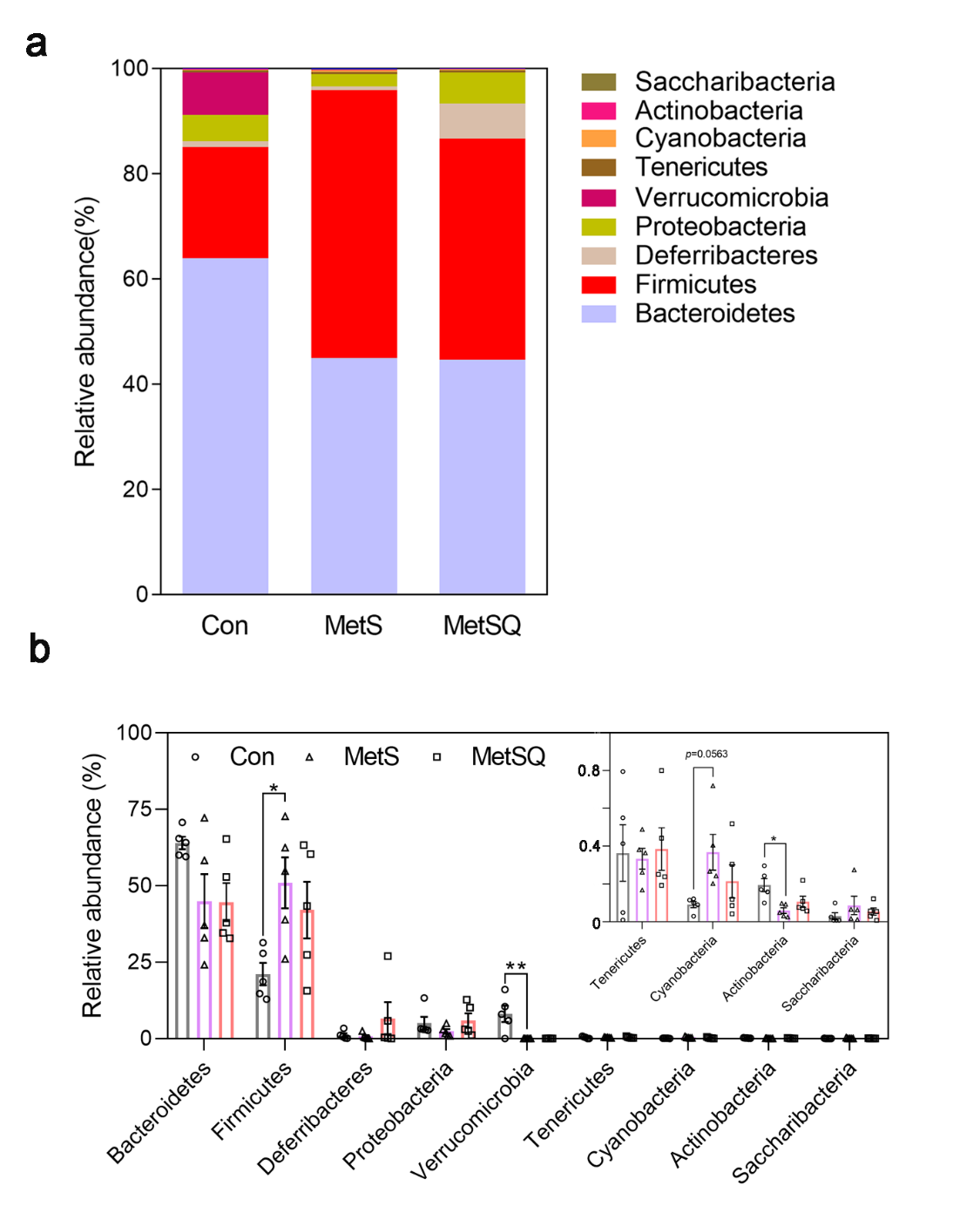


Figure S9. The (a) gut microbiota structure and (b) statistical analyses at phylum level. Related to Figure 5. Data are shown as mean ± s.e.m, n=5 in each group, **p*< 0.05, ***p*< 0.01.


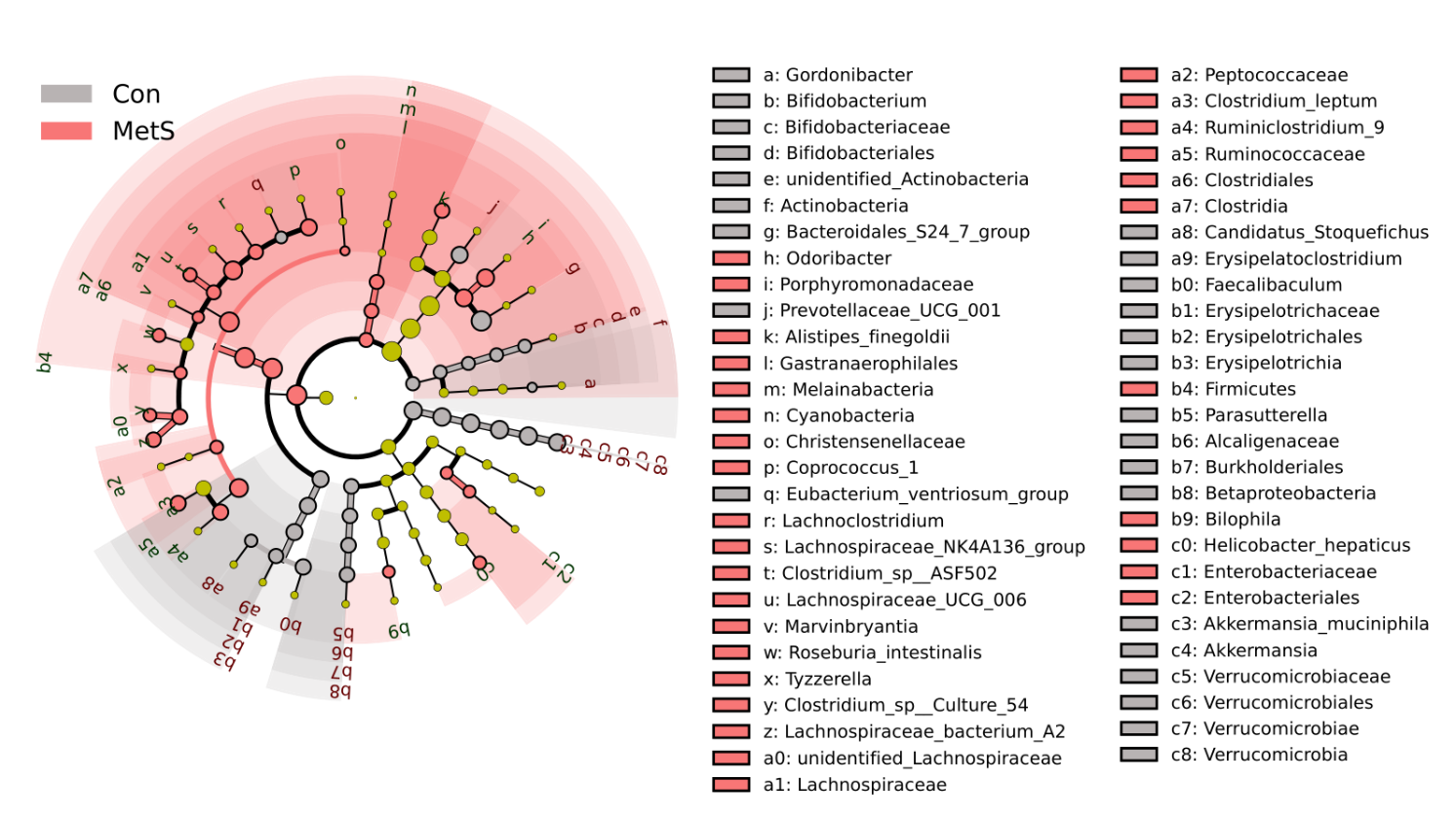


Figure S10. LEfSe of gut microbiota between Con and MetS groups. Related to Figure 5, n=5 in each group.


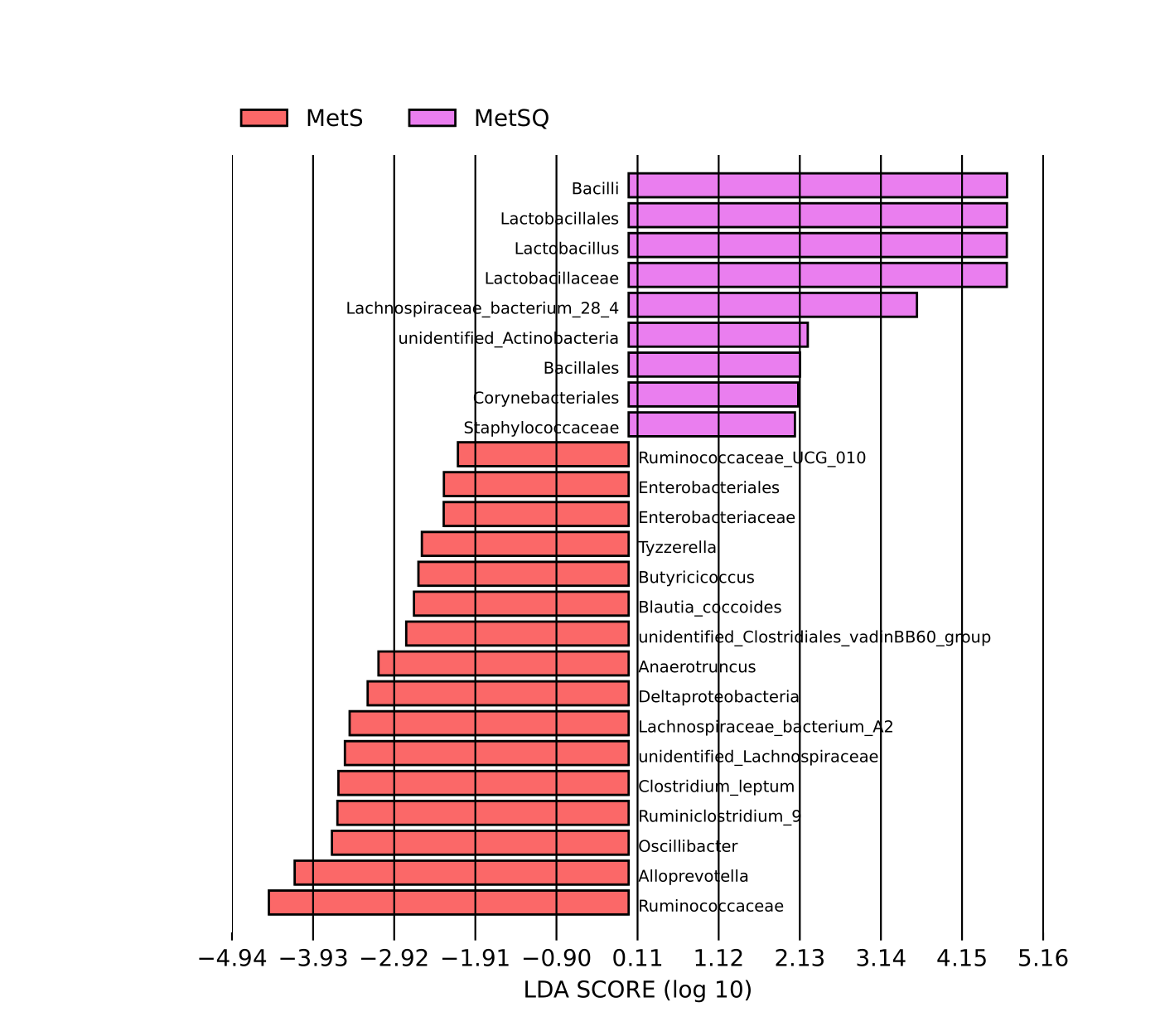


Figure S11. LDA of gut microbiota between MetS and MetSQ groups. Related to Figure 5, n=5 in each group.


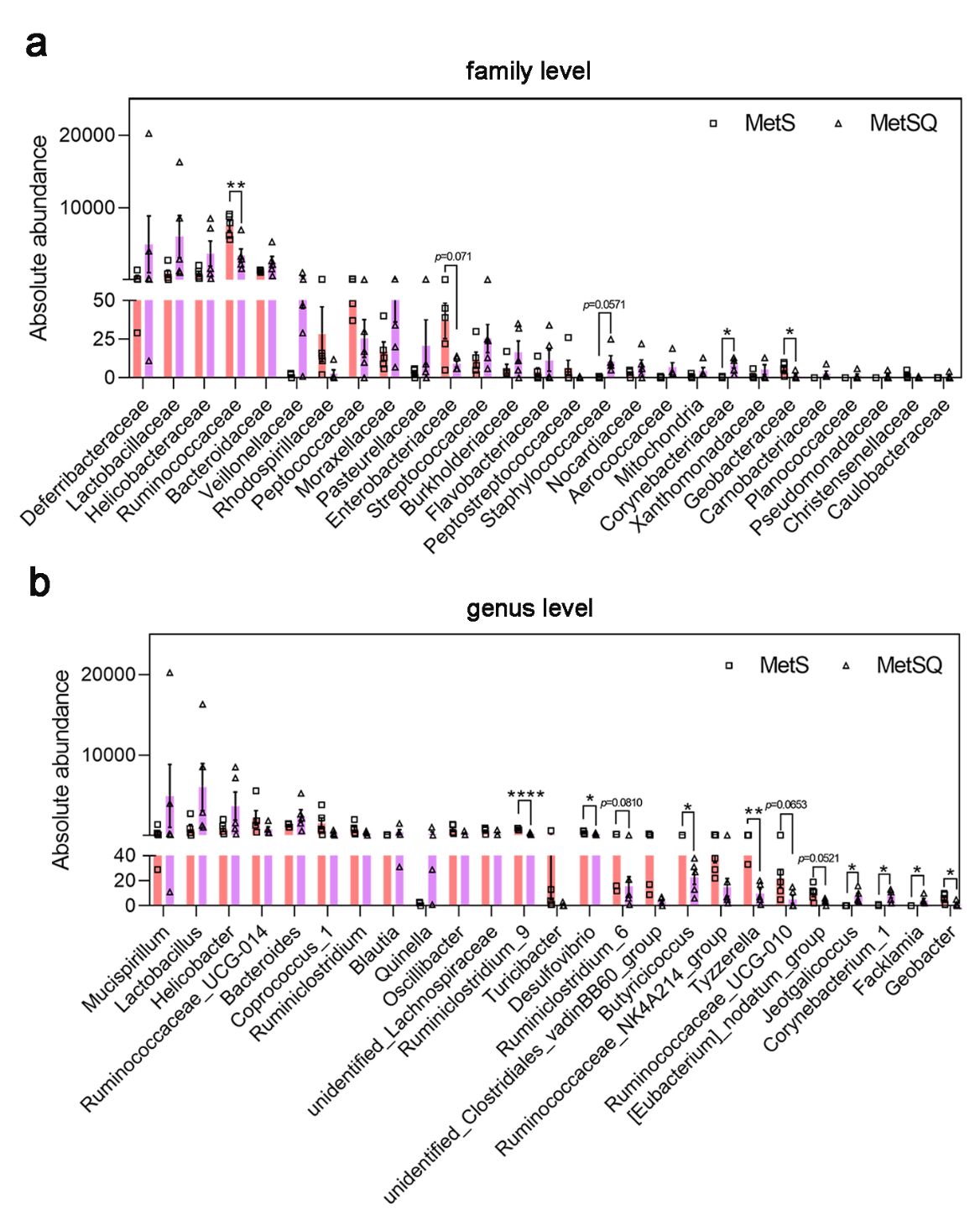


Figure S12. The absolute abundances of gut microbiota at (a) family level and (b) genus level. Related to Figure 5. Data are shown as mean ± s.e.m, n=5 in each group, **p*< 0.05, ***p*< 0.01, **** *p*< 0.0001.


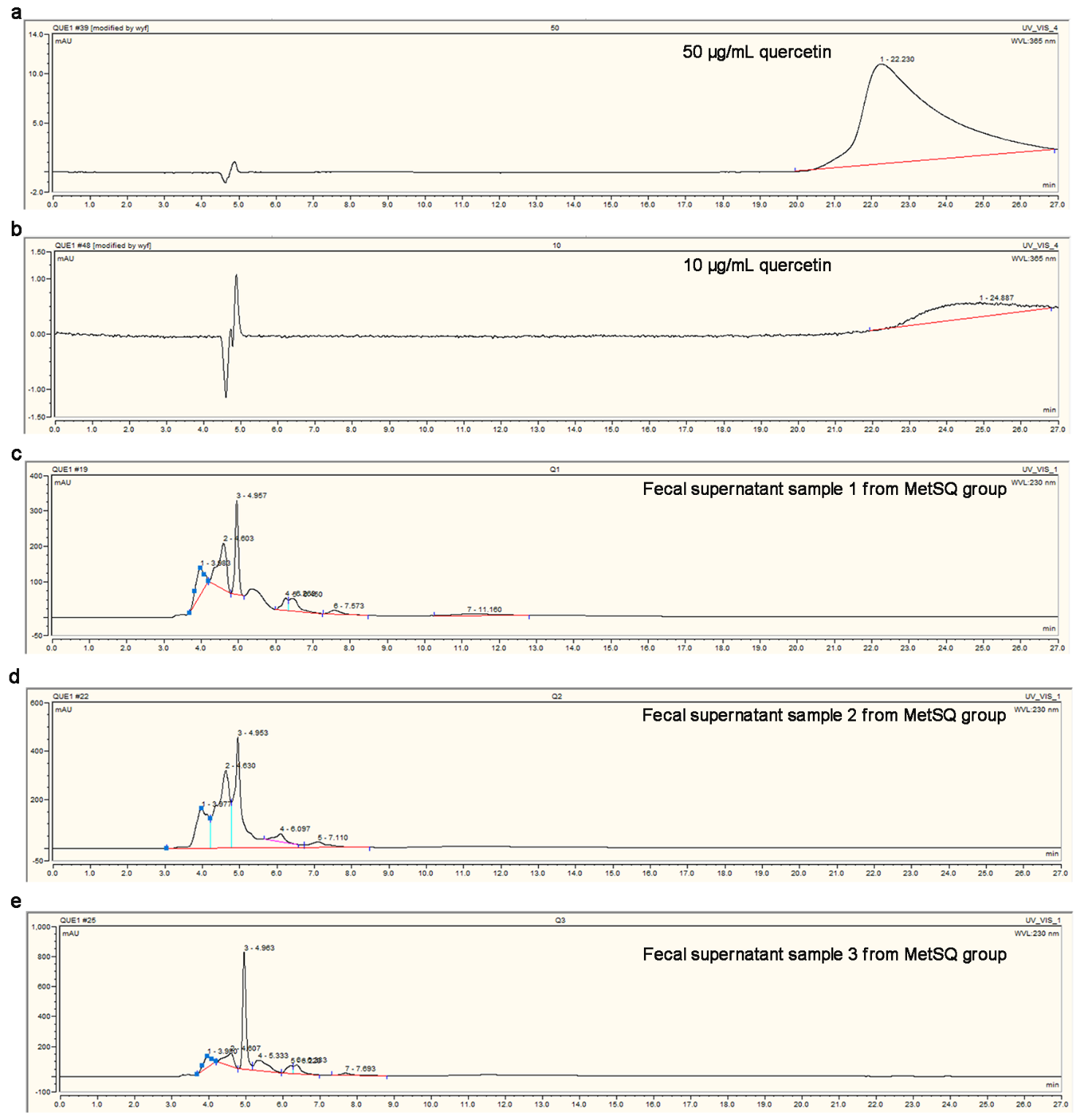


Figure S13. HPLC chromatogram for quercetin in fecal supernatant. (a, b) HPLC chromatogram for 50 and 10 μg/mL standard quercetin. (c-e) HPLC chromatogram for fecal supernatant samples of MetSQ group.


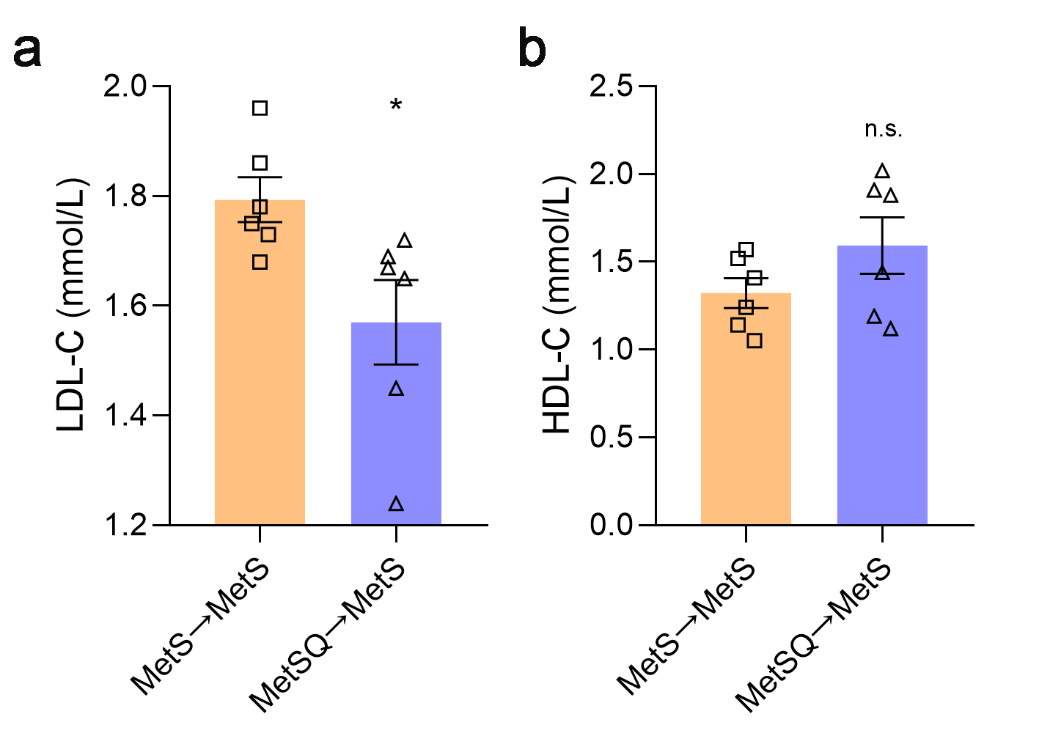


Figure S14. The (a) LDL-C and (b) HDL-C levels in serum after FMT. Related to Figure 6. All values are shown as mean ± s.e.m, n=6 in each group, **p*<0.05, n.s., non-significant.


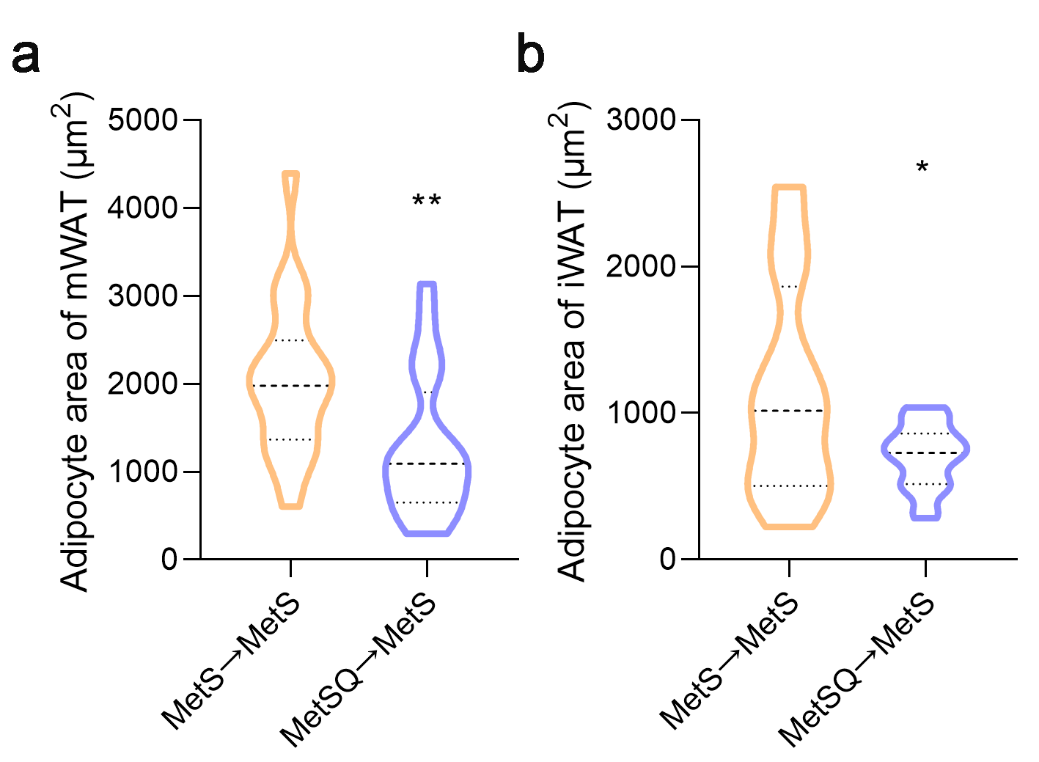


Figure S15. The average adipocyte area of (a) mWAT and (b) iWAT after FMT. Related to Figure 6. All values are shown as mean ± s.e.m, n=6 in each group, **p*< 0.05, ***p*< 0.01.


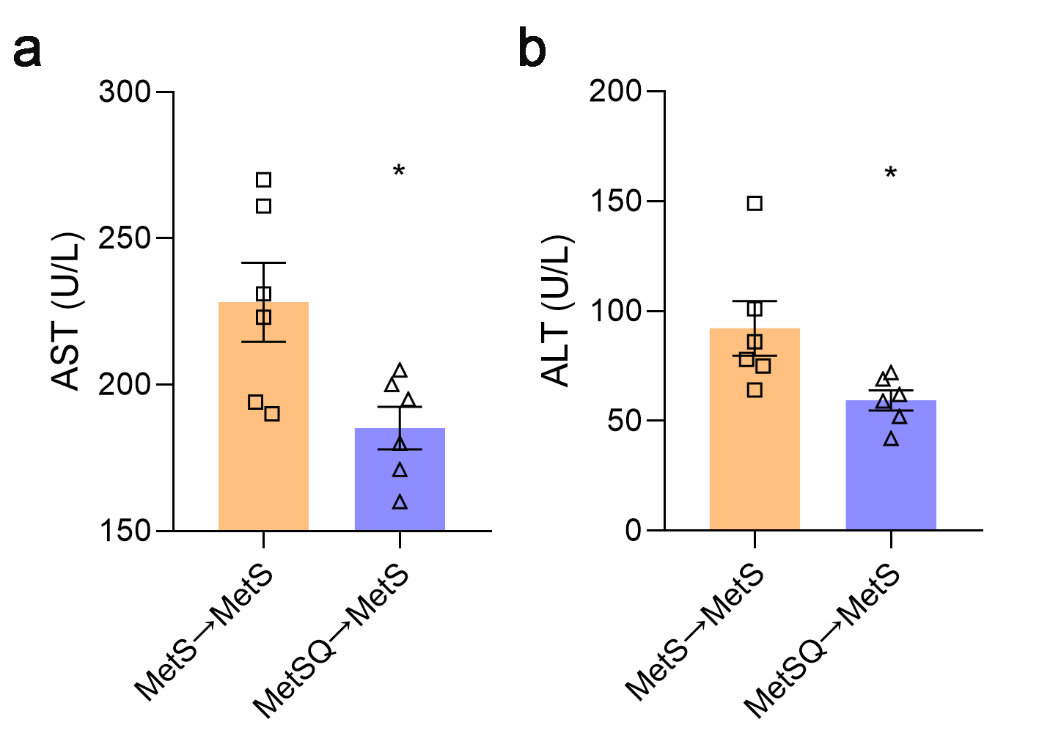


Figure S16. The (a) AST and (b) ALT levels in serum after FMT. Related to Figure 6. All values are shown as mean ± s.e.m, n=6 in each group, **p*< 0.05.


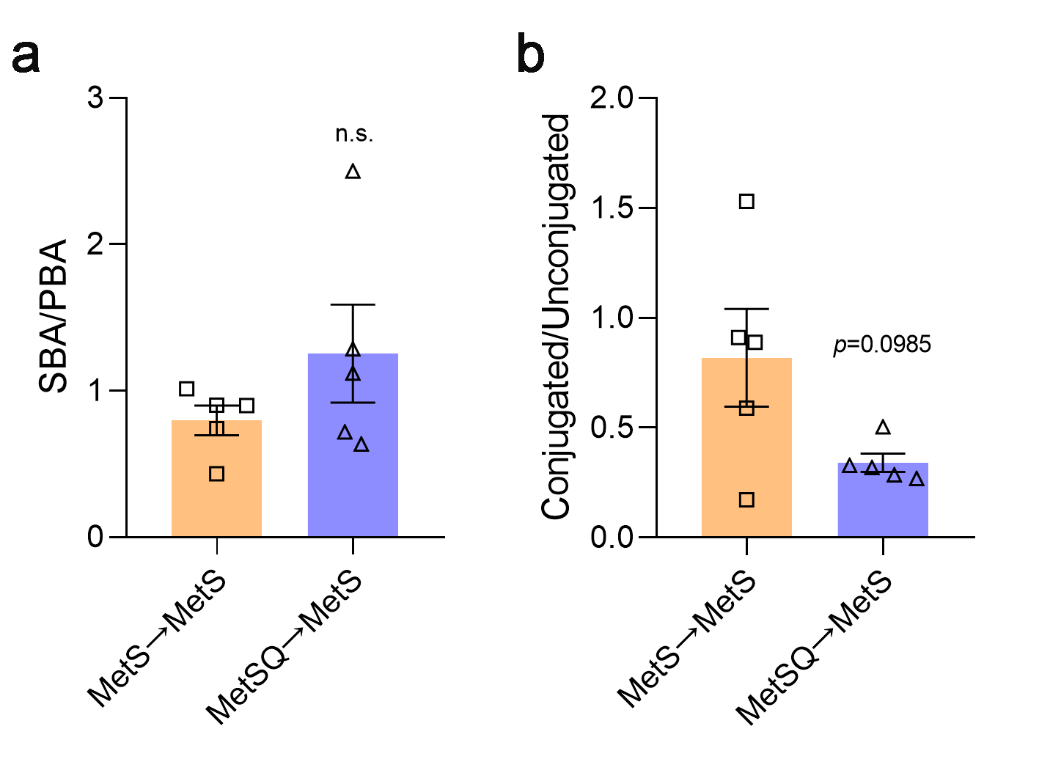


Figure S17. The ratio of (a) SBA concentration to PBA concentration and (b) conjugated BA concentration to unconjugated BA concentration after FMT. Related to Figure 9. All values are shown as mean ± s.e.m, n=5 in each group, n.s., non-significant.


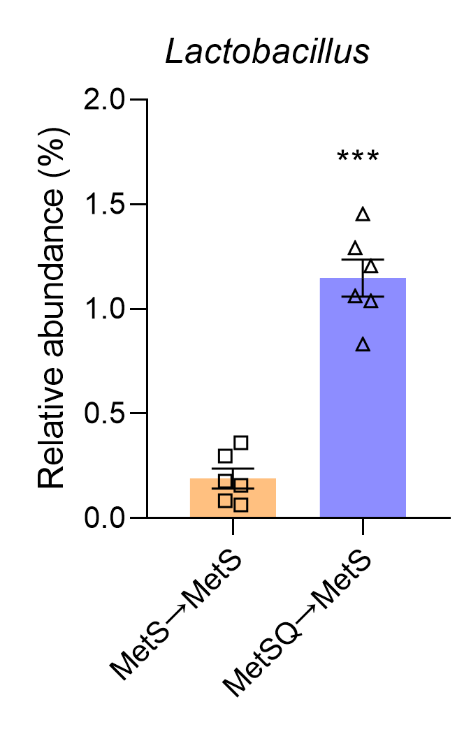


Figure S18. The relative abundance of *Lactobacillus* after FMT. Related to Figure 9. All values are shown as mean ± s.e.m, n=6 in each group, *****p*< 0.0001.

**Table S1. Primers used for quantitative real-time PCR**

| **Gene** | **Forward primer (5’-3’)** | **Reverse primer (5’-3’)** |
| --- | --- | --- |
| *β-actin* | GGCTGTATTCCCCTCCATCG | CCAGTTGGTAACAATGCCATGT |
| *Pgc1α* | TTCATCTGAGTATGGAGTCGCT | GGGGGTGAAACCACTTTTGTAA |
| *Ucp1*  *Tgr5* | TGCCAGGCAAGCTGAAACTC  TGCTTCTTCCTAAGCCTACTACT | GTGAACCCGACAACTTCCGAA  CTGATGGTTCCGGCTCCATAG |
| *Cyp7a1* | GTTGTCCAAAGGAGGTTCACC | GCTGTGGTAGTGAGCTGTTG |
| *Cyp7b1*  *Cyp8b1* | GCCATGCCAAGATAAGGAAGC  TGAGCACCAGTTCTTTTGCATAG | GGAGCCACGACCCTAGATG  CACGGGGATGTCTTCACGG |
| *Cyp27a1* | CGGGCAAGTGCAGCACATA | GCACAGGAGAGTACGGAGG |
